# Supplementary material for: Overcoming small-bandgap charge recombination in visible and NIR-light-driven hydrogen evolution by engineering the polymer photocatalyst structure
Source: Nat Commun. 2024 Jan 24;15:707. doi: 10.1038/s41467-024-45085-6 (PMC10808228; doi:10.1038/s41467-024-45085-6)
Supplement: Supplementary file 1 — Supplementary Information [file 41467_2024_45085_MOESM1_ESM.pdf]

Supplementary Information

Overcoming Small-Bandgap Charge Recombination in Visible and NIR-Light-Driven Hydrogen Evolution by Engineering the Polymer Photocatalyst Structure

Mohamed Hammad Elsayed[1,2,3,4], *Mohamed Abdellah*[5,6,7], *Ahmed Zaki Alhakemy*[8], *Islam M. A. Mekhemer*[4], *Ahmed Esmail A. Aboubakr* [9,10,11], *Bo-Han Chen*[12], *Amr Sabbah*[3, 13], *Kun-Han Lin*[4], *Wen-Sheng Chiu*[14], *Sheng-Jie Lin*[14], *Che-Yi Chu*[14], *Chih-Hsuan Lu*[12], *Shang-Da Yang*[12], *Mohamed Gamal Mohamed*[15], *Shiao-Wei Kuo*[15], *Chen-Hsiung Hung*[11], *Li-Chyong Chen*[3, 13, 16], *Kuei-Hsien Chen*[2, 3], *Ho-Hsiu Chou**[4]

[1]Department of Chemistry, Faculty of Science, Al-Azhar University, Nasr City 11884, Cairo, Egypt.

[2]Institute of Atomic and Molecular Sciences, Academia Sinica, Taipei, 10617, Taiwan.

[3]Center for Condensed Matter Sciences, National Taiwan University, Taipei, 10617, Taiwan.

[4]Department of Chemical Engineering, National Tsing Hua University, Hsinchu 300044, Taiwan.

[5]Department of Chemistry, United Arab Emirates University, Al Ain P.O. Box 15551, United Arab Emirates.

[6]Department of Chemistry, Qena Faculty of Science, South Valley University, 83523 Qena, Egypt.

[7]Chemical Physics and NanoLund, Lund University, 22100 Lund, Sweden.

[8]Chemistry Department, Faculty of Science, Al-Azhar University, Assiut, 71542, Egypt.

[9]Sustainable Chemical Science and Technology, Taiwan International Graduate Program, Taiwan.

[10]Department of Applied Chemistry, National Yang Ming Chiao Tung University, Hsinchu 30010, Taiwan.

[11]Institute of Chemistry, Academia Sinica, 128 Sec 2 Academia Rd., Nankang, Taipei, 11529, Taiwan.

[12]Institute of Photonics Technologies, National Tsing Hua University, Hsinchu, 30013 Taiwan.

[13]Center of Atomic Initiative for New Materials, National Taiwan University, Taipei 10617, Taiwan.

[14]Department of Chemical Engineering, National Chung Hsing University. Taichung 40227, Taiwan.

[15]Department of Materials and Optoelectronic Science, Center for Functional Polymers and Supramolecular Materials, National Sun Yat-Sen University, Kaohsiung 804, Taiwan

[16]Department of Physics, National Taiwan University, Taipei 10617, Taiwan.

*Corresponding Authors

E-mail: hhchou@mx.nthu.edu.tw

Keywords: *π*-linker effect; near-infrared photocatalyst; polymer dots photocatalysts, visible-light; hydrogen evolution; ITIC-polymers

Contents **Page**

- **Synthesis procedures of polymers** 6
  - Synthesis of compound 3 (5-bromo-1H-indene-1,3(2H)-dione). 6
  - Synthesis of compound 5 (IC-Br) (2-(5(6)-bromo-3-oxo-2,3-dihydro-1H-inden-1-ylidene) malononitrile). 7
  - Synthesis of compound 7 (Br-ITIC-Br). 7
  - Synthesis of compound 10 (Br-BTIC-Br). 9
  - Synthesis of PITIC-Ph and PBTIC-Ph polymers 11
  - Synthesis of PITIC-Th and PBTIC-Th polymers 11
  - Synthesis of PITIC-ThF and PBTIC-ThF polymer 11
- **Supplementary Fig. 1.** Synthetic procedures for the IC-Br and Br-ITIC-Br. 9
- **Supplementary Fig. 2.** Synthetic procedures for the IC-Br and Br-BTIC-Br. 10
- **Supplementary Fig. 3.** Synthetic procedures for PITIC-Ph, PITIC-Th, and PITIC-ThF polymers. 13
- **Supplementary Fig. 4.** Synthetic procedures for PBTIC-Ph, PBTIC-Th, and PBTIC-ThF polymers. 14
- **Supplementary Fig. 5.** ^{1}H NMR spectrum for 5-bromo-1H-indene-1, 3(2H)-dione 15
- **Supplementary Fig. 6.** ^{1}H NMR spectrum for 2-(5(6)-bromo-3-oxo-2, 3-dihydro-1H-inden-1-ylidene) malononitrile 16
- **Supplementary Fig. 7.** ^{1}H NMR spectrum for Br-ITIC-Br 17
- **Supplementary Fig. 8.** ^{1}H NMR spectrum for Br-BTIC-Br 18
- **Supplementary Fig. 9.** ^{1}H NMR spectrum of PITIC-Ph 19
- **Supplementary Fig. 10.** ^{1}H NMR spectrum of PITIC-Th 20
- **Supplementary Fig. 11.** ^{1}H NMR spectrum of PITIC-ThF 21
- **Supplementary Fig. 12.** ^{1}H NMR spectrum of PBTIC-Ph 22
- **Supplementary Fig. 13.** ^{1}H NMR spectrum of PBTIC-Th 23
- **Supplementary Fig. 14.** ^{1}H NMR spectrum of PBTIC-ThF 24
- **Supplementary Fig. 15.** High resolution mass spectra of IC-Br monomer 25
- **Supplementary Fig. 16.** High resolution mass spectra of Br-ITIC-Br monomer 26
- **Supplementary Fig. 17.** High resolution mass spectra of Br-BTIC-Br monomer 27
- **Supplementary Fig. 18.** Thermogravimetric analysis for all polymers 28
- **Supplementary Fig. 19.** (a) FTIR and (b) XPS data of materials 29
- **Supplementary Fig. 20.** The high-resolution XPS of elements 30
- **Supplementary Fig. 21.** The high-resolution XPS of other elements 31

- **Supplementary Fig. 22.** The high-resolution XPS of F1s peak 32
- **Supplementary Fig. 23.** Synthesis of Pdot structure 33
- **Supplementary Fig. 24.** UV-vis abs of monomers ad their polymers 34
- **Supplementary Fig. 25.** UV-vis abs of polymers in THF or water 35
- **Supplementary Fig. 26.** The HOMO levels of polymers were determined by a photoelectron spectrometer 36
- **Supplementary Fig. 27.** Water contact angles for all polymer materials 37
- **Supplementary Fig. 28.** Emission spectra of the light source used in the hydrogen evolution reaction experiment. 38
- **Supplementary Fig. 29.** Effect of H_2PtCl_6 and Pt nanoparticles on HER 39
- **Supplementary Fig. 30.** HER of PITIC-ThF under NIR light and Visible light using the same light source (Xe lamp) 40
- **Supplementary Fig. 31.** HER stability test of PITIC-ThF Pdot for 24 h 41
- **Supplementary Fig. 32.** UV-Vis absorption, FTIR spectrum, and DLS before and after H2 evolution experiment 42
- **Supplementary Fig. 33.** Energy level diagrams of the all-polymers in an acidic and neutral medium 44
- **Supplementary Fig. 34.** F−H and S−H bonding distance of polymers 45
- **Supplementary Fig. 35.** The frontier orbitals of PBTIC series 46
- **Supplementary Fig. 36.** The frontier orbitals of PITIC series 46
- **Supplementary Fig. 37.** The relative energy levels along the linker-acceptor dihedral angle scanning of ground state (S_0) and excited states (S_1, S_2 and S_3) 47
- **Supplementary Fig. 38.** 2D GIWAXS patterns for polymers 48
- **Supplementary Fig. 39.** PL, EIS, Photocurrent response, and TRPL measurements of polymers 50
- **Supplementary Fig. 40.** Cryo-TEM micrograph, and cryo-electron diffraction of the polymers 52
- **Supplementary Fig. 41.** Transient absorption spectra for PITIC-ThF Pdot with 0.1M AA with a power of 0.9 μW 54
- **Supplementary Fig. 42.** Transient absorption spectra for PITIC-Th Pdot with Pt, AA, or Pt+AA with a power of 0.9 μW 55
- **Supplementary Fig. 43.** Transient absorption spectra for PITIC-ThF Pdot with Pt, AA, or Pt+AA with a power of 10 μW 57
- **Supplementary Fig. 44.** Absorptivity of PITIC-X Pdots 58
- **Supplementary Fig. 45.** Effect of Triton on the PITIC-ThF Pdots 59
- **Supplementary Fig. 46.** Particle size distributions measured by dynamic light scattering (DLS) of all presented polymers nanoparticles in water 60
- **Supplementary Table 1.** Physical properties of polymer dots 61
- **Supplementary Table 2.** Physical and optical properties of polymers 61

- **Supplementary Table 3.** The residual Pd contents of polymers 62
- **Supplementary Table 4.** Determined molecular wight of all polymers 62
- **Supplementary Table 5.** Comparative studies of our developed polymer photocatalyst versus other polymer photocatalysts 63
- **Supplementary Table 6.** Comparative studies of our developed polymer photocatalyst versus other photocatalysts 66
- **Supplementary Table 7.** The excited-state and charge-transfer properties for each polymer 67
- **Supplementary Table 8.** The equivalent circuit fitted results of EIS data 68
- **Supplementary Table 9.** The equivalent circuit fitted results of EIS data 68
- **Supplementary Table 10.** The equivalent circuit fitted results of EIS data 68
- **Supplementary References** 69

Synthesis procedures of polymers

All reagents were obtained from commercial suppliers and used without further purification. 4-Bromophthalic Anhydride (compound **1**), Ethyl acetoacetate (compound **2**), malononitrile (compound **4**), and the other chemicals and solvents were purchased from Matrix and Alfa Aesar. Compound **6** (**IDTT-CHO**) (Dithieno[2,3-d:2,3-d]-s-indaceno[1,2-b:5,6-b]dithiophene-2,8-dicarboxaldehyde, 6,6,12,12-tetrakis(4-hexylphenyl)-6,12-dihydro), compound **B-Ph-B** (1,4-bis(4,4,5,5-tetramethyl-1,3,2-dioxaborolan-2-yl)benzene), compound **Sn-Th-Sn** (2,5-Bis(trimethylstannyl)thiophene), and compound **Sn-ThF-Sn** (3,4-difluoro-2,5-bis-trimethylstannanylthiophene) were purchased from Derthon Optoelectronic Materials Science Technology Co Ltd. The compound IC-Br, the monomer Br-ITIC-Br, and the PITIC-Ph, PITIC-Th, PITIC-ThF polymers were synthesized according to supplementary Figs. 1 and 2.

Synthesis of compound 3 (5-bromo-1H-indene-1,3(2H)-dione). Compound **1** (4-bromophthalic anhydride) ((5 g, 22 mmol), acetic anhydride (12 mL) and triethylamine (6.5 mL) was added to a two-necked round bottom flask under a nitrogen atmosphere, then the compound **2** (ethyl acetoacetate) (3.15 mL) was added quickly under argon. The solution color will be changed from orange to red after the addition of compound **2**. The mixture was stirred at room temperature for 22 h, then ice-water (8.5 g) and concentrated HCl (8 mL) were added to the mixture followed by the addition of 5M HCl (35 mL). After that, the mixture was stirred at 80 °C for 15 min. Then, the crude product was cooled to room temperature, water was added, and the mixture was extracted with dichloromethane (DCM). The organic phase was taken, and the solvent evaporated under reduced pressure. The dried solid was dissolved in a minimum volume of acetone and cooled to –20 °C. The

product was collected by filtration and then dried under high vacuum to give a material which was obtained as a brown solid. ^{1}H-NMR (500 MHz, CD_2Cl_2): δ 8.1(s, 1H), 7.94 (d, 1H), 7.83 (d, 1H), 3.23(s, 2H).

Synthesis of compound 5 (IC-Br) (2-(5(6)-bromo-3-oxo-2,3-dihydro-1H-inden-1-ylidene) malononitrile). Compound 3 (2.33 g, 10.35 mmol) and malononitrile (1.37 g, 20.7 mmol) were mixed in 40 mL ethanol in a 250 mL single neck round bottom flask and stirred for 30 min at room temperature. Then, anhydrous sodium acetate (1.28 g, 15.52 mmol) was added to the reaction, and the mixture was stirred at room temperature for 2 h. After the reaction 40 mL water was added, and the mixture was stirred at room temperature for half an hour. Then, concentrated HCl was dropped into the mixture to acidify the mixture with pH = 2. The precipitate was filtered and washed with water many times. The crude product was further purified by flash column chromatography to afford title compound 3 as yellow solid (0.38 g, yield: 29.4%). 1H NMR (500 MHz, CDCl3) δ 8.75 (s, 1H), 8.49 (d, 1H), 8.1 (s, 1H), 7.97 (m, 1H), 7.82 (d, 1H), 3.72 (d, 2H). HR-FD-MS: m/z: 273.9590 for $C_{12}H_5BrN_2O$. Elemental analysis found C 52.31%, N 10.06 %, H 2.29 %, O 6.06 %.

Synthesis of compound 7 (Br-ITIC-Br). Compound 5 (558 mg, 2.04 mmol) and Compound 6 (400 mg, 0.38 mmol) were added to a 250 ml two-necked round bottom flask, after being rinsed with a mild stream of nitrogen for ten minutes, then anhydrous chloroform (100 mL) was added to the flask. Finally, pyridine (2 mL) was added to the reaction, the mixture turned green gradually. Then, the reaction was placed in an oil bath at 65 ºC stirred and refluxed for 12 hours. After the reaction was completed, it was cooled to room temperature. The crude product was poured into methanol (400 mL), then the

precipitate was filtered and purified by flash column (DCM/Hexane (1:1)). 1H NMR (500 MHz, CDCl3) δ 8.85 (d, 1H), 8.79 (dd, 0.5H), 8.52 (dd, 0.5H), 8.21 (d, 1H), 7.99 (dd, 0.5H), 7.85-7.82 (m, 1H), 7.74 (dd, 0.5H), 7.62 (m, 1H), 7.19-7.10 (dd, 8H), 2.56-2.53 (m, 4H), 1.60-1.52 (m, 4H), 1.32 (dq, 12H), 0.84 (t, 6H). HR-FD-MS: m/z: 1582.3536 for $C_{94}H_{80}Br_2N_4O_2S_4$. Elemental analysis found C 71.55 %, N 3.55 %, H 5.19 %, O 2.24 %, S 6.86%.

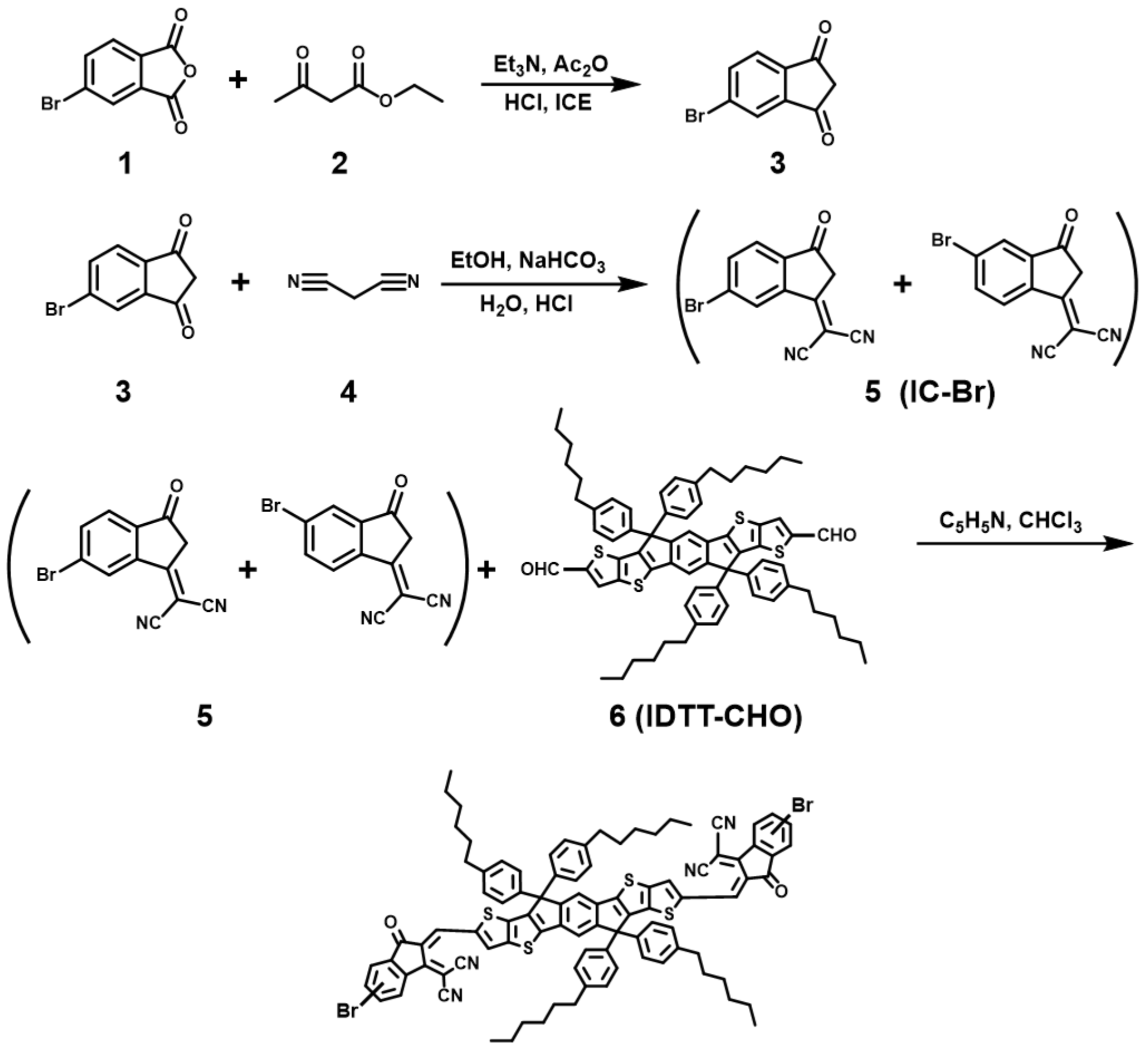

7 (Br-ITIC-Br)

Supplementary Fig. 1. Synthetic procedures for the IC-Br (isomers containing Br atom at either 5 or 6 positions) and the monomers of Br-ITIC-Br (isomers containing Br atom at either 5 or 6 positions).

Synthesis of compound 10 (Br-BTIC-Br). Compound 5 (558 mg, 2.04 mmol) and Compound 9 (400 mg, 0.38 mmol) were added to a 250 ml two-necked round bottom flask, after being rinsed with a mild stream of nitrogen for ten minutes, then anhydrous chloroform (100 mL) was added to the flask. Finally, pyridine (2 mL) was added to the

reaction, the mixture turned green gradually. Then, the reaction was placed in an oil bath at 65 ºC stirred and refluxed for 12 hours. After the reaction was completed, it was cooled to room temperature. The crude product was poured into methanol (400 mL), then the precipitate was filtered and purified by flash column (DCM/Hexane (1:1)). 1H NMR (500 MHz, CDCl3) δ δ 9.14 (s, 1H), 8.68 (d, J = 5.7 Hz, 0.5H), 7.96 (s, 0.5H), 7.83 – 7.65 (m, 2H), 4.90 – 4.67 (m, 4H), 3.22 (t, J = 7.6 Hz, 4H), 2.10 – 2.00 (m, 2H), 1.90-1.80 (m, 2H), 1.41 – 0.92 (m, 52H), 0.81-0.60 (m, 20H), 0.68 (m, 6H). HR-FD-MS: m/z: 1534.4006 for $C_{82}H_{8}Br_{2}N_{8}O_{2}S_{5}$. Elemental analysis found C 64.67 %, N 6.71 %, H 5.95 %, O 3.22 %, S 9.55%.

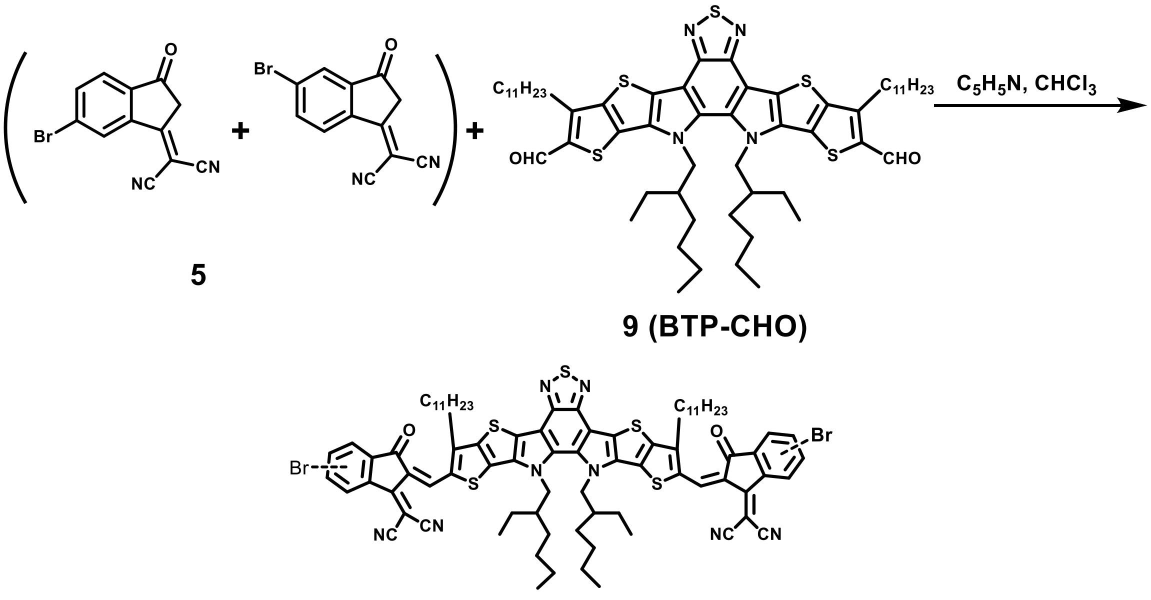

5

9 (BTP-CHO)

10 (Br-BTIC-Br)

Supplementary Fig. 2. Synthetic procedures for the IC-Br (isomers containing Br atom at either 5 or 6 positions) and the monomers of Br-BTIC-Br (isomers containing Br atom at either 5 or 6 positions).

Synthesis of PITIC-Ph and PBTIC-Ph polymers: PITIC-Ph and PBTIC-Ph were prepared by Suzuki–Miyaura coupling polymerization. Monomer Br-ITIC-Br (158.5 mg 0.1 mmol) or Br-BTIC-Br (153.2 mg 0.1 mmol), respectively, monomer B-Ph-B (33 mg, 0.1 mmol), Na_2CO_3 (79 mg, 0.75 mmol), tetra-n-butylammonium bromide (1.6 mg, 0.005 mmol), and $Pd(PPh_3)_4$ (9.0 mg, 0.008 mmol), toluene (10 mL), and water (2.5 mL) were injected into a sealed tube (Recently, We have opted for the refluxing technique in a round bottom flask (more safety) over using a sealed tube which give the same results). The mixture was degassed by bubbling with N_2 for 30 min and then heated at 120 °C for 72 h. After cooling to room temperature, bromobenzene was added and then the sealed tube was heated at 120 °C for 6 h, followed by the addition of phenylboronic acid and heating at 120 °C for another 6 h. The mixture was cooled to room temperature and poured into MeOH. The precipitate was collected through membrane filtration. Purification of the polymer was performed through Soxhlet extraction with MeOH and hexane. Finally, the polymer was dissolved in hot $CHCl_3$, concentrated, and then precipitated in MeOH. The polymer was collected and dried under vacuum. The PITIC-Ph and PBTIC-Ph were isolated as powders in 63% and 57% yield, respectively.

Synthesis of PITIC-Th and PBTIC-Th polymers: PITIC-Th and PBTIC-Th were prepared by Stille coupling polymerization. Monomer Br-ITIC-Br (158.5 mg 0.1 mmol) or Br-BTIC-Br (153.2 mg 0.1 mmol), respectively, monomer Sn-Th-Sn (41 mg, 0.1 mmol), $Pd(PPh_3)_4$ (9.0 mg, 0.008 mmol), and anhydrous toluene (10 mL) were added to a sealed tube (Recently, We have opted for the refluxing technique in a round bottom flask (more safety) over using a sealed tube which give the same results). The mixture was degassed by bubbling with N_2 for 30 min and then stirred at 100 °C for 24 h. After cooling to room

temperature, the mixture was poured into MeOH. The precipitate was collected through membrane filtration. Purification of the polymer was performed through Soxhlet extraction with MeOH and hexane. Finally, the polymer was dissolved in hot $CHCl_3$, concentrated, and then precipitated in MeOH. The polymer was collected and dried under vacuum. The PITIC-Th and PBTIC-Th were isolated as powders in 51% and 54% yield, respectively.

Synthesis of PITIC-ThF and PBTIC-ThF polymer: PITIC-Th and PBTIC-ThF were prepared by the same synthetic method of PITIC-Th mentioned above. Monomer Br-ITIC-Br (158.5 mg 0.1 mmol) or Br-BTIC-Br (153.2 mg 0.1 mmol), respectively, monomer Sn-ThF-Sn (44.6 mg, 0.1 mmol), $Pd(PPh_3)_4$ (9.0 mg, 0.008 mmol), and anhydrous toluene (10 mL) were added to a sealed tube (Recently, We have opted for the refluxing technique in a round bottom flask (more safety) over using a sealed tube which give the same results). Then follow the same procedure above. The PITIC-ThF and PBTIC-ThF were isolated as powders in 52% and 55% yield, respectively.

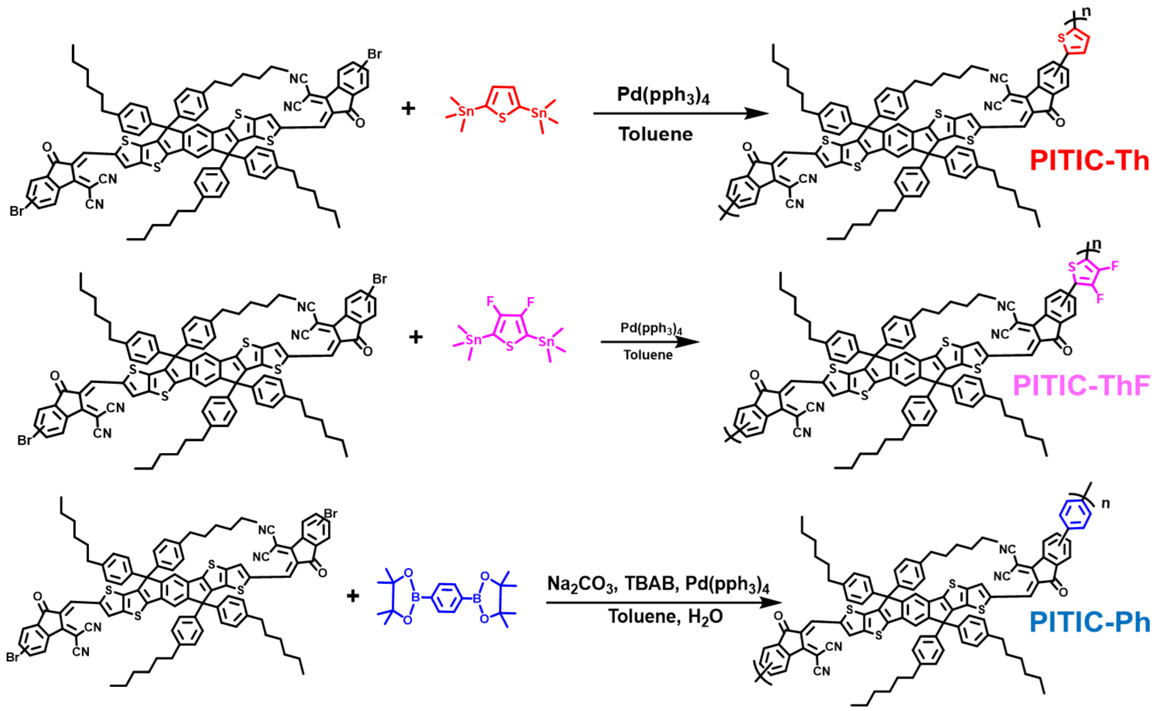

Supplementary Fig. 3. Synthetic procedures for PITIC-Ph, PITIC-Th, and PITIC-ThF polymers.

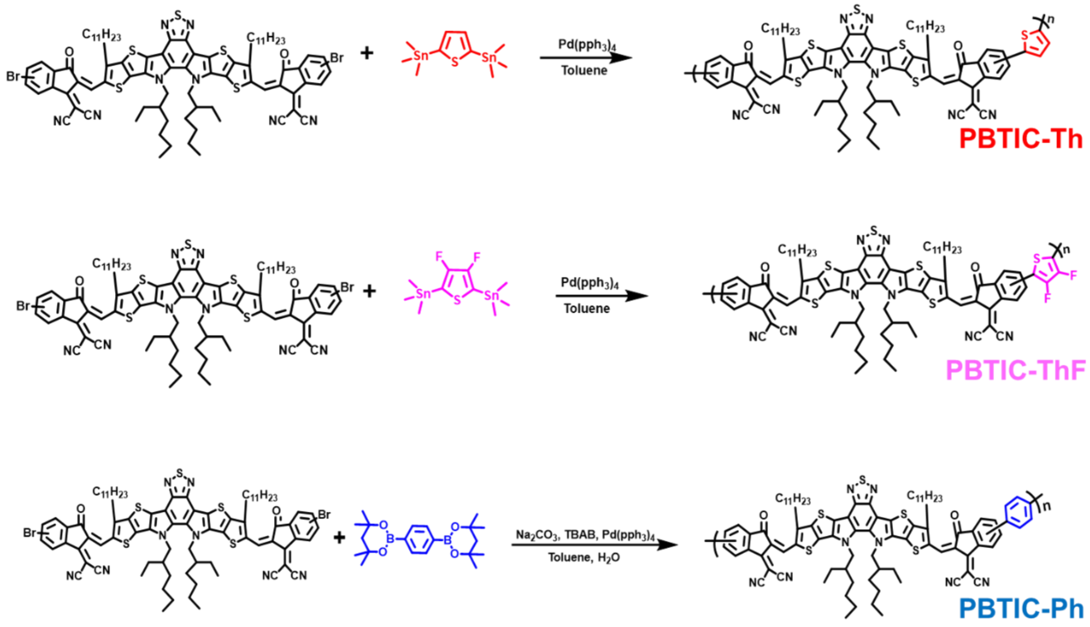

Supplementary Fig. 4. Synthetic procedures for PBTIC-Ph, PBTIC-Th, and PBTIC-ThF polymers.

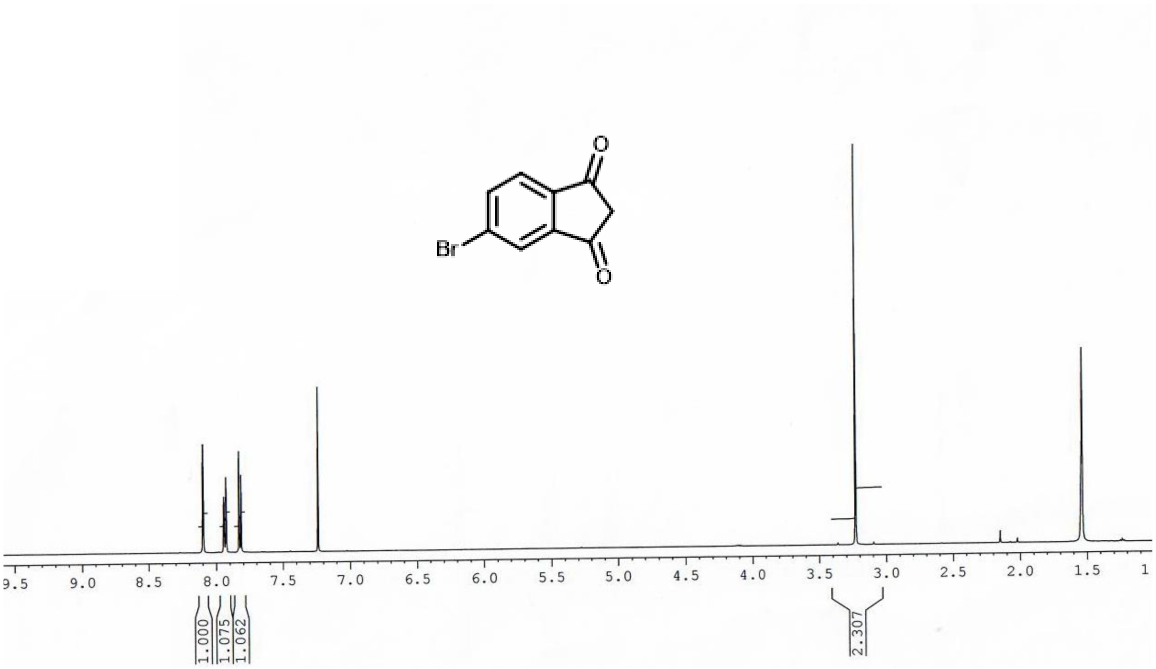

Supplementary Fig. 5. ^{1}H NMR spectrum for 5-bromo-1H-indene-1,3(2H)-dione measured in $CDCl_3$.

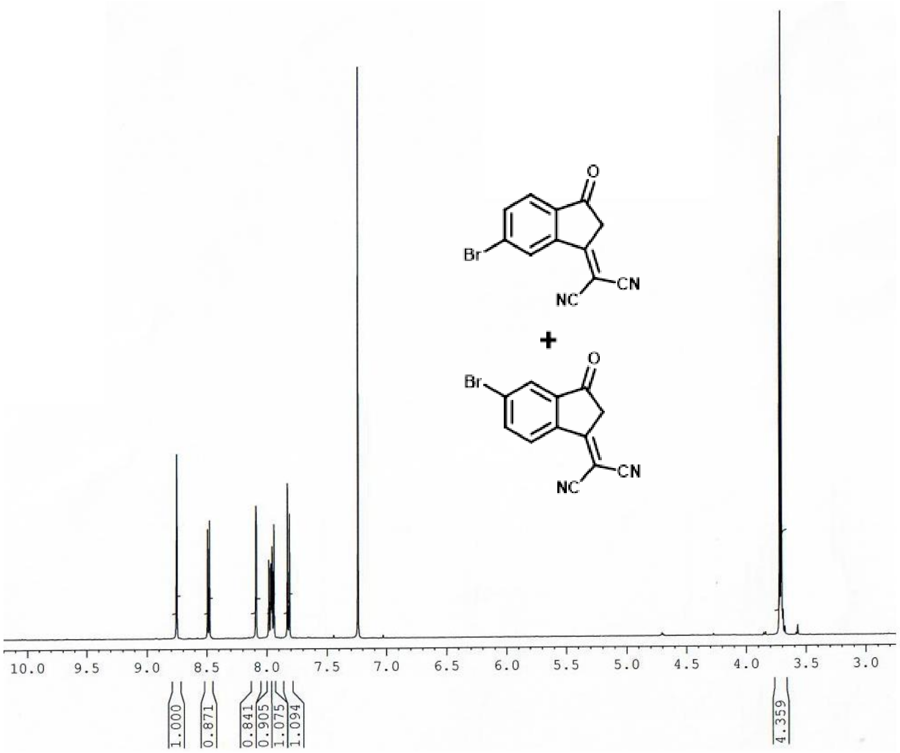

Supplementary Fig. 6. ^{1}H NMR spectrum for 2-(5(6)-bromo-3-oxo-2,3-dihydro-1H-inden-1-ylidene) malononitrile measured in $CDCl_3$.

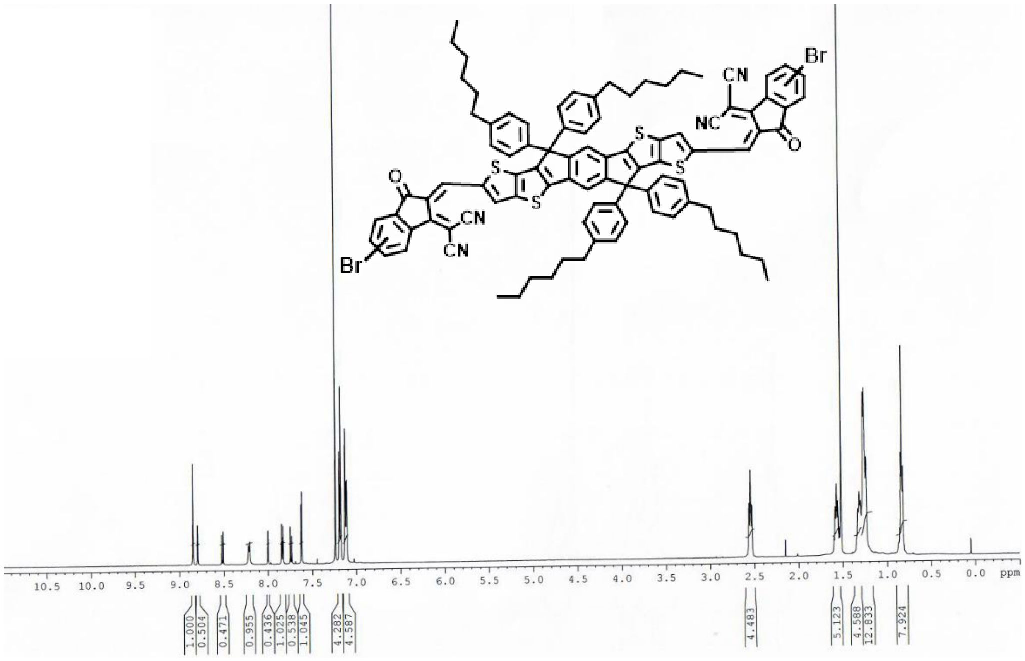

Supplementary Fig. 7. ^{1}H NMR spectrum for Br-ITIC-Br measured in $CDCl_3$.

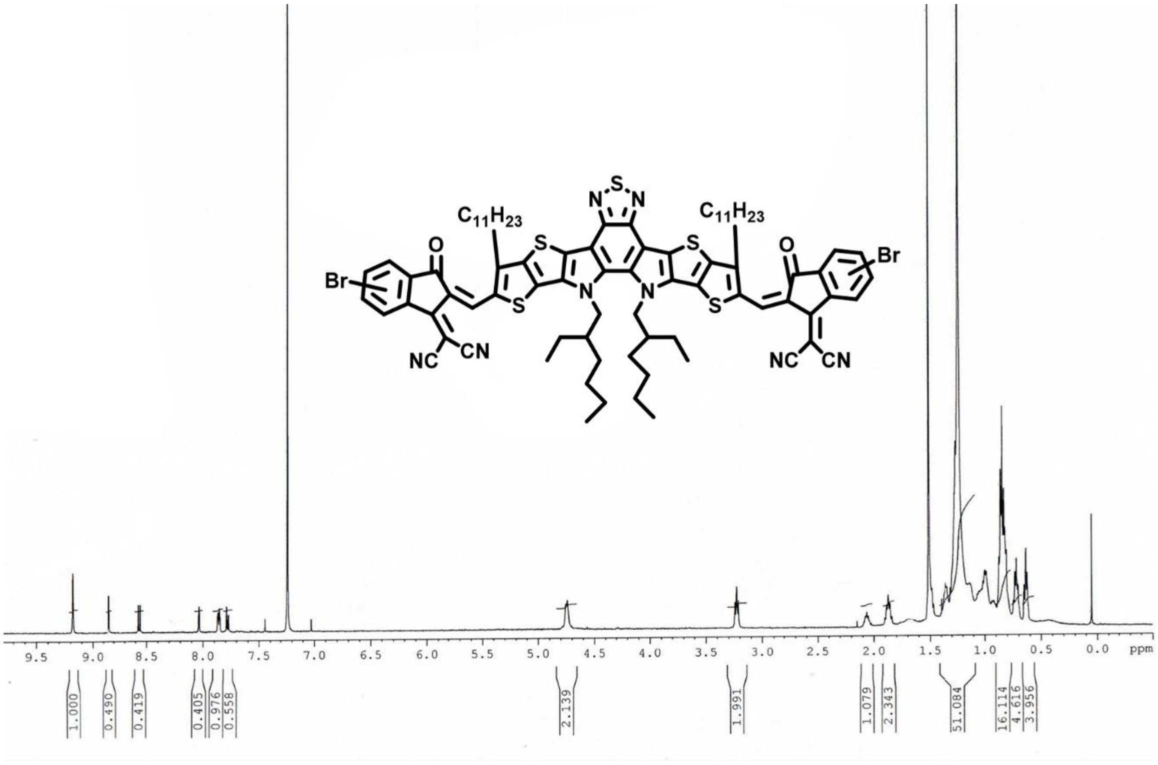

Supplementary Fig. 8. ^{1}H NMR spectrum for Br-BTIC-Br measured in $CDCl_3$.

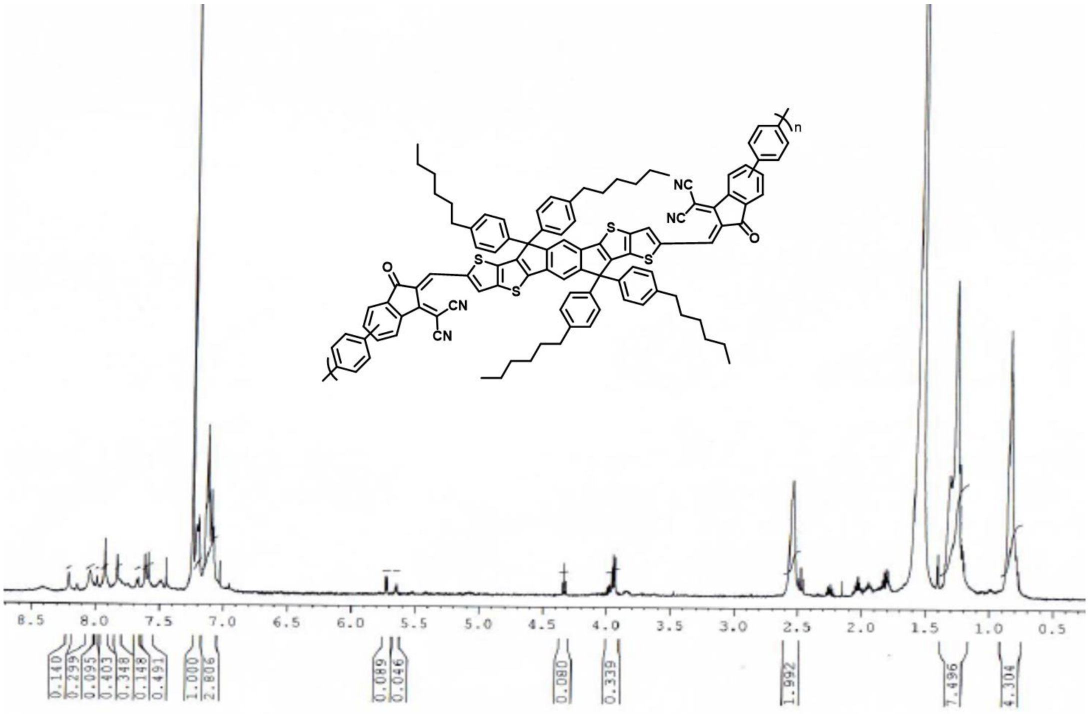

Supplementary Fig. 9. ^{1}H NMR spectrum of PITIC-Ph in $CDCl_3$.

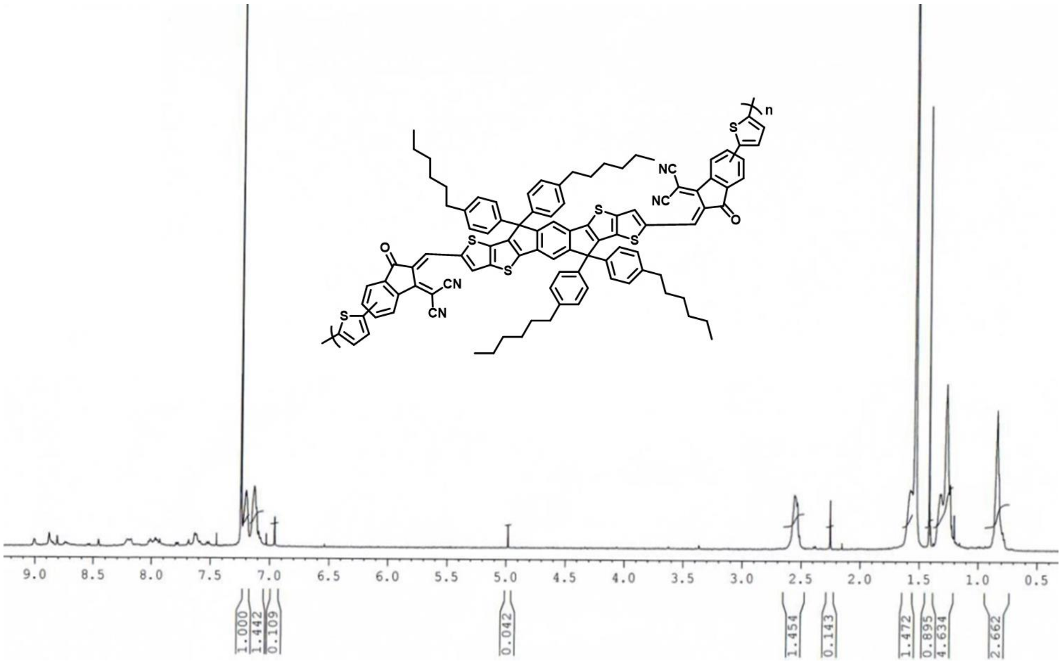

Supplementary Fig. 10. ^{1}H NMR spectrum of PITIC-Th in $CDCl_3$.

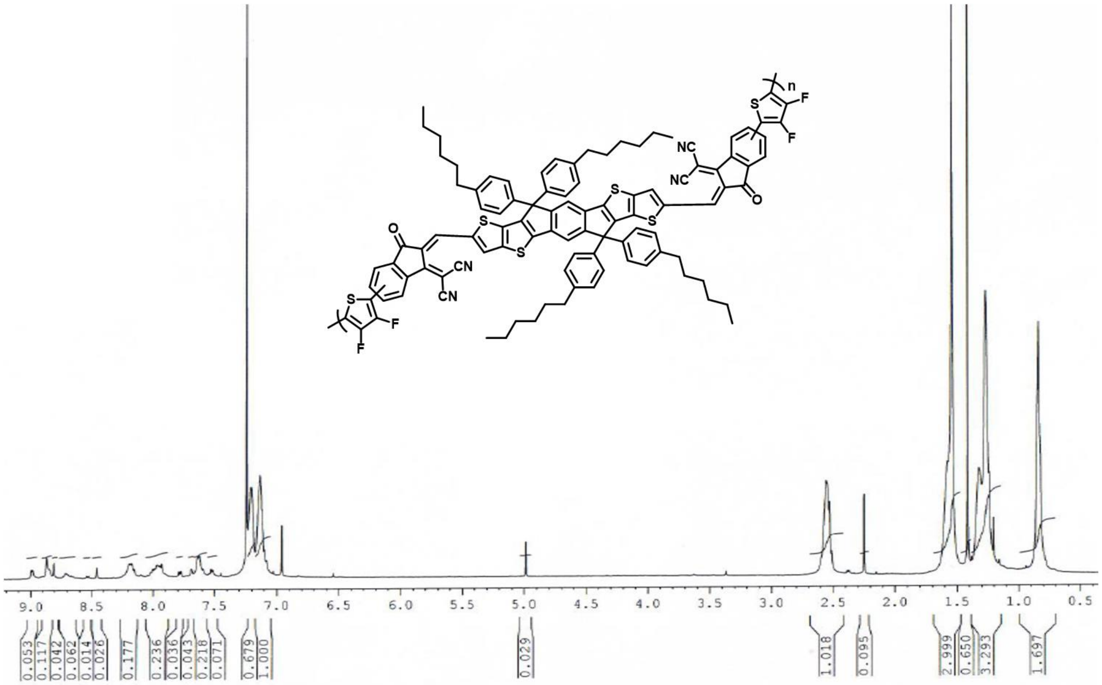

Supplementary Fig. 11. ^{1}H NMR spectrum of PITIC-ThF in $CDCl_3$.

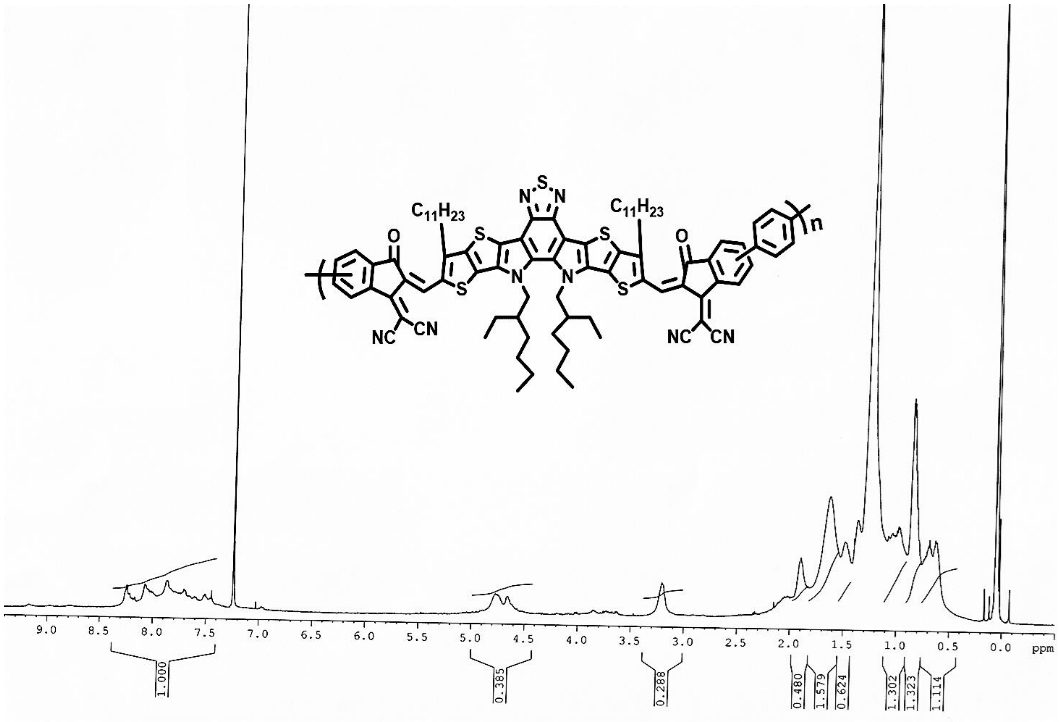

Supplementary Fig. 12. ^{1}H NMR spectrum of PBTIC-Ph in $CDCl_3$.

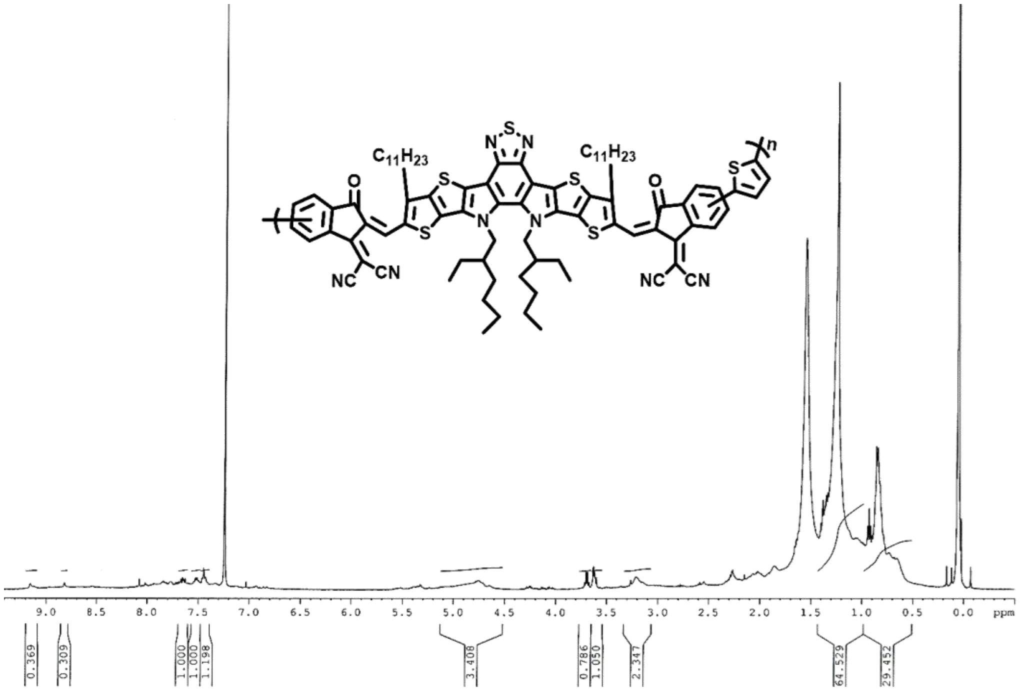

Supplementary Fig. 13. ^{1}H NMR spectrum of PBTIC-Th in $CDCl_3$.

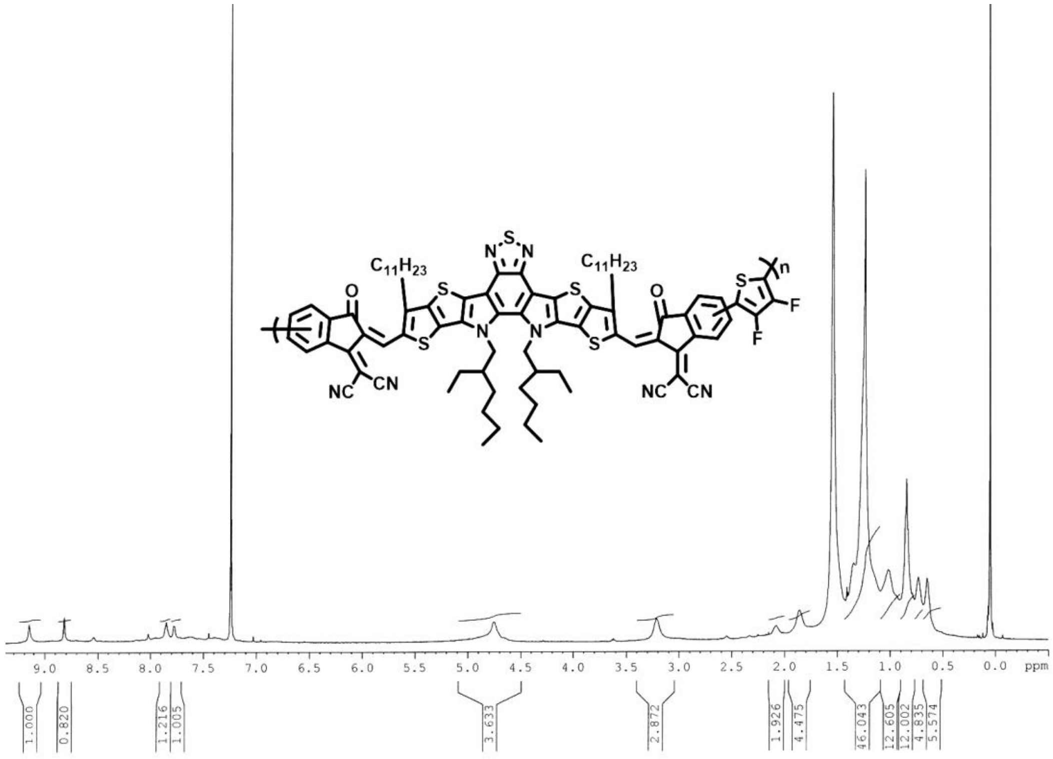

Supplementary Fig. 14. ^{1}H NMR spectrum of PBTIC-ThF in $CDCl_3$.

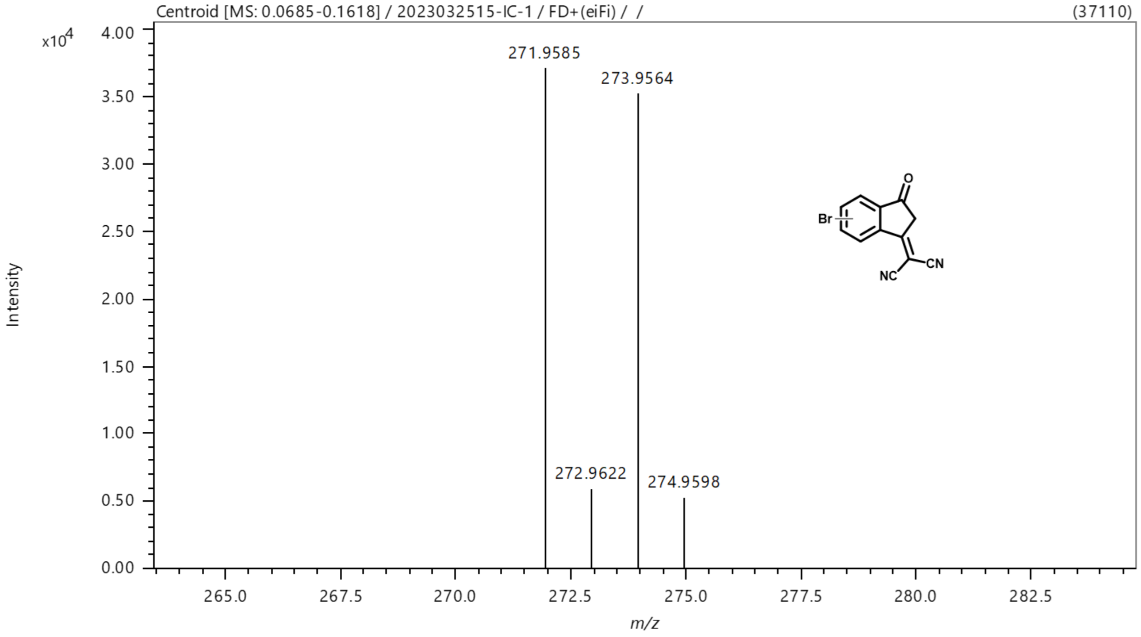

Supplementary Fig. 15. High resolution mass spectra of IC-Br monomer. HR-FD-MS: m/z: 273.9590. The chemical formula and theoretical molecular weight of IC-Br are C12H5BrN2O and 273.09, respectively.

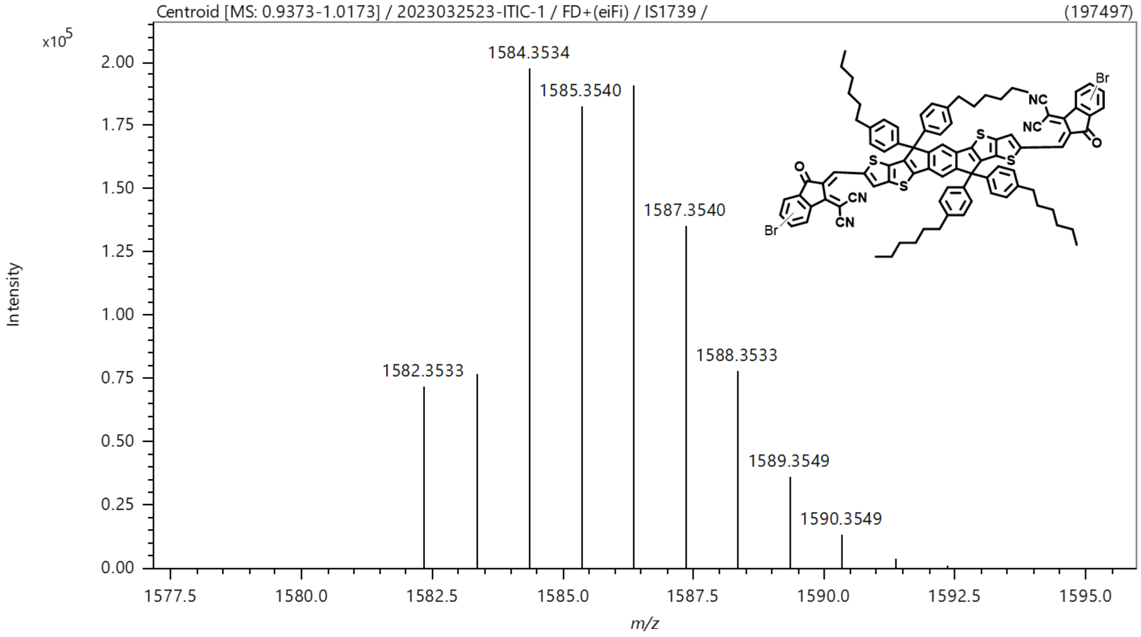

Supplementary Fig. 16. High resolution mass spectra of Br-ITIC-Br monomer. HR-FD-MS: m/z: 1582.3536. The chemical formula and theoretical molecular weight of Br-ITIC-Br are $C_{94}H_{80}Br_2N_4O_2S_4$ and 1585.75, respectively.

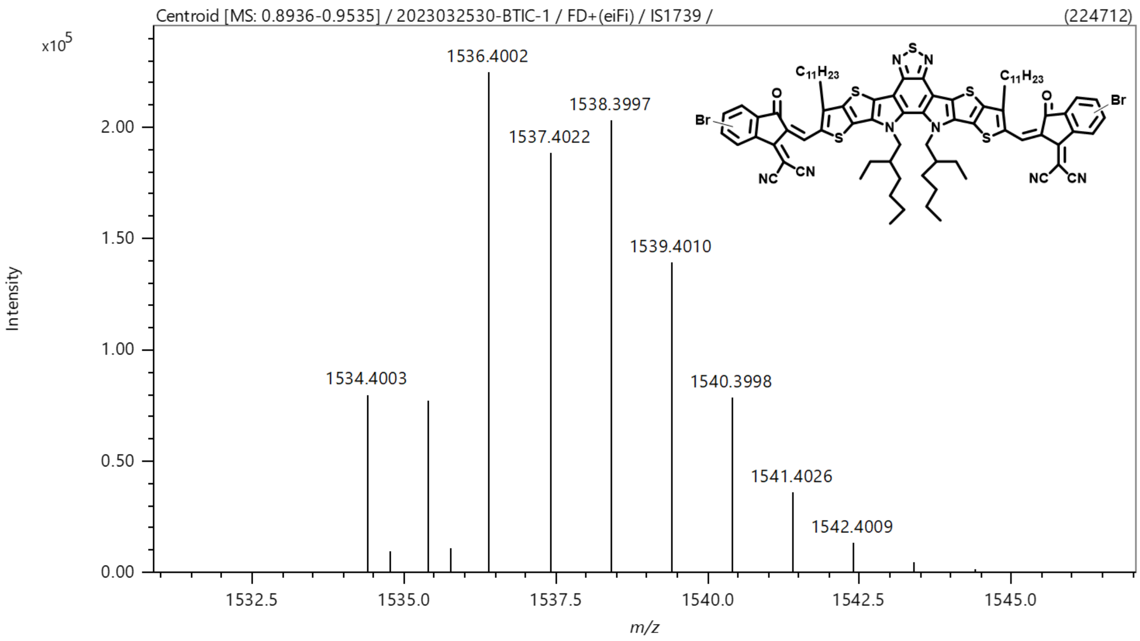

Supplementary Fig. 17. High resolution mass spectra of Br-BTIC-Br monomer. HR-FD-MS: m/z: 1534.4006. The chemical formula and theoretical molecular weight of Br-BTIC-Br are C82H88Br2N8O2S5 and 1537.77, respectively.

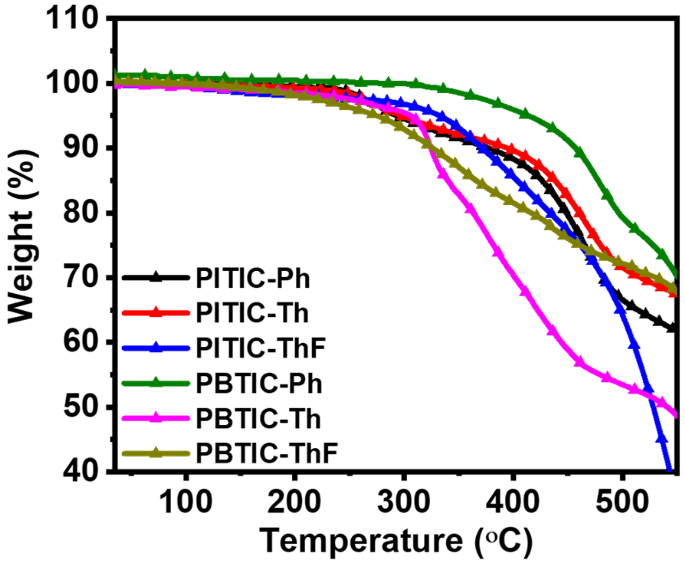

Supplementary Fig. 18. Thermogravimetric analysis for all polymers. The TGA curves show that both PITIC-X- and PBTIC-X-based polymers have good thermal stability, with high decomposition temperatures ranging from 290−413 °C.

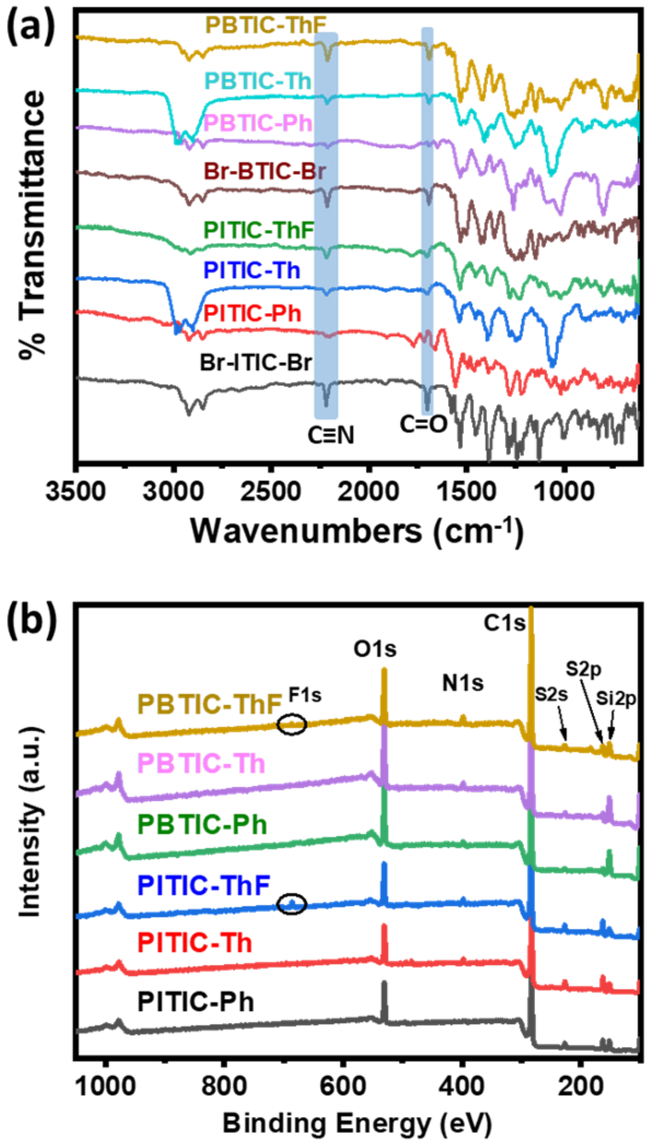

Supplementary Fig. 19. (a) FTIR and (b) XPS data of PITIC-X, and PBTIC-X based polymers and their starting materials. The FTIR results have characteristic absorption peaks at 2,200 cm^{-1} and 1,700 cm^{-1}, which correspond to the appearance of C≡N and C=O groups, respectively; these are present in all polymers.

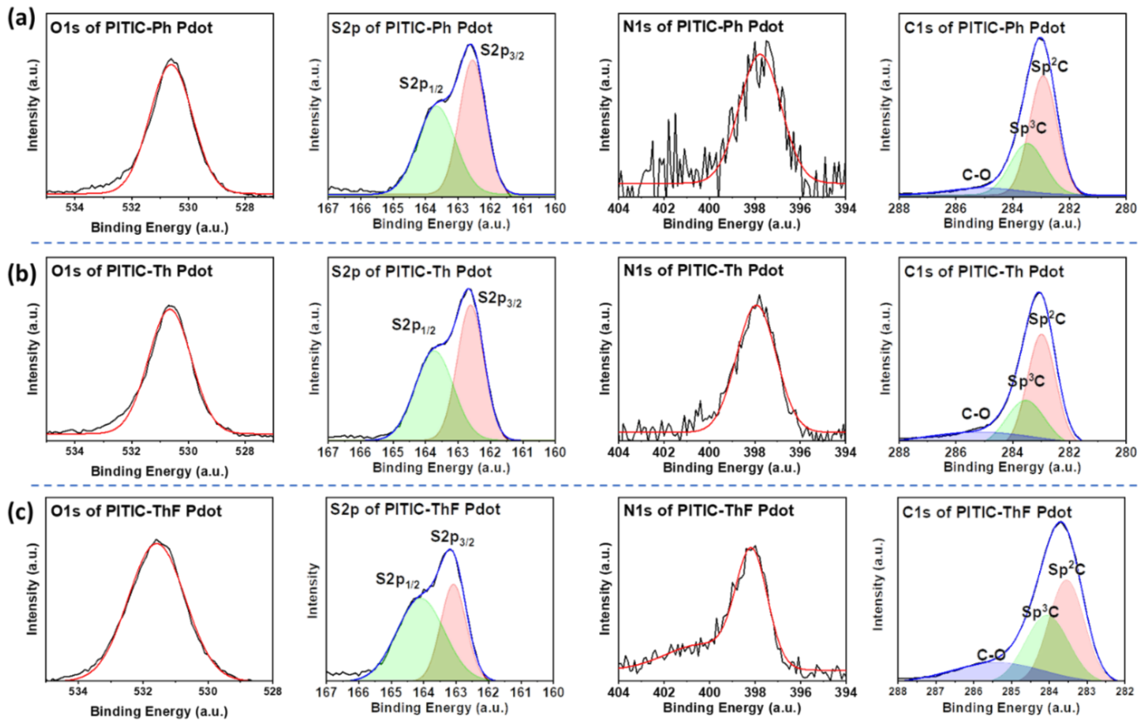

Supplementary Fig. 20. The high-resolution XPS of C1s, O1s, S2p and N1s peaks for (a) PITIC-Ph, (b) PITIC-Th, (c) PITIC-ThF. The surface chemical composition of the polymers was analyzed by XPS methods to investigate the surface electronic structure and bonding configuration.

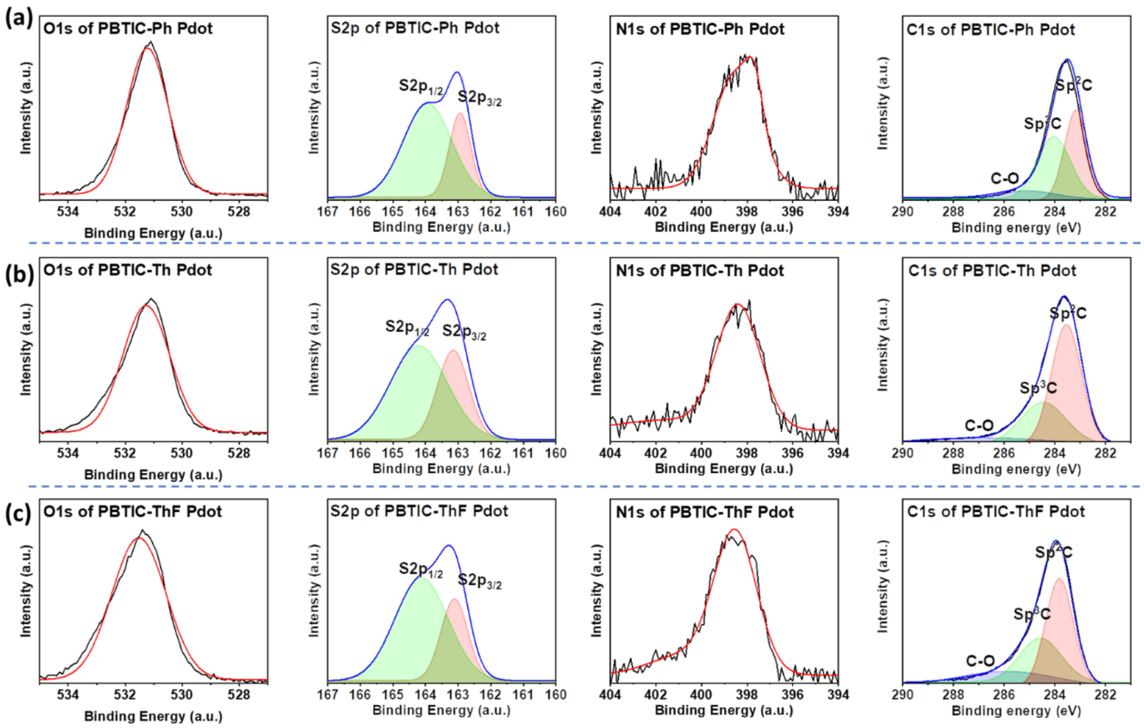

Supplementary Fig. 21. The high-resolution XPS of C1s, O1s, S2p and N1s peaks for (a) PBTIC-Ph, (b) PBTIC-Th, (c) PBTIC-ThF.

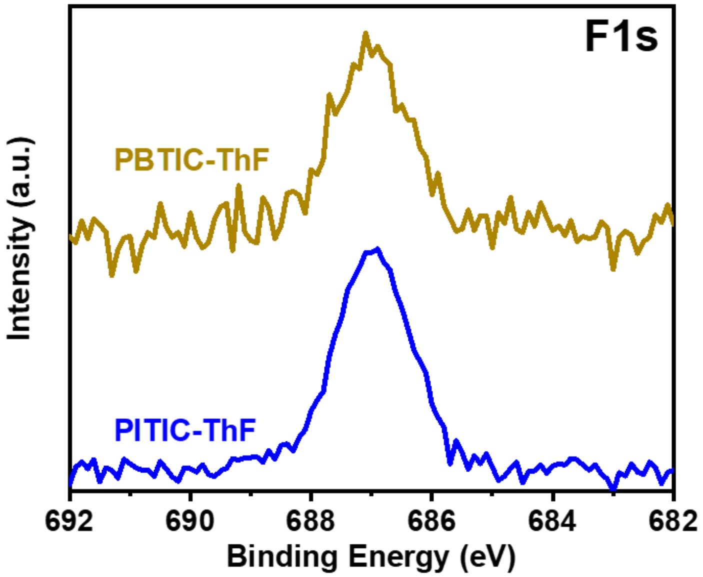

Supplementary Fig. 22. The high-resolution XPS of F1s peak for PBTIC-ThF and PITIC-ThF. The XPS spectra reveal that the major constituent elements in the six polymers are C, N, O, and S, while F is detected only in the PITIC-ThF and PBTIC-ThF polymers**.**

These results indicate the purity of the prepared polymers from any other elements that may originate during the preparation process. Supplementary Figs. 20, and 21 show the high-resolution C 1s, S 2p, N 1s, and O 1s XPS peaks of all six polymers, and Supplementary Figs. 22 shows the high-resolution F 1s XPS peaks for PBTIC-ThF and PITIC-ThF. The C 1s spectra can be deconvoluted into three peaks corresponding to sp^2 carbon bonding (sp^2 C), sp^3 carbon bonding (sp^3 C), and the carbon in C=O bonding. Furthermore, the S 2p spectra of the PITIC-Th(ThF) and PBTIC-Th (ThF) polymers can be deconvoluted into two peaks corresponding to S $2p_{3/2}$ and S $2p_{1/2}$.

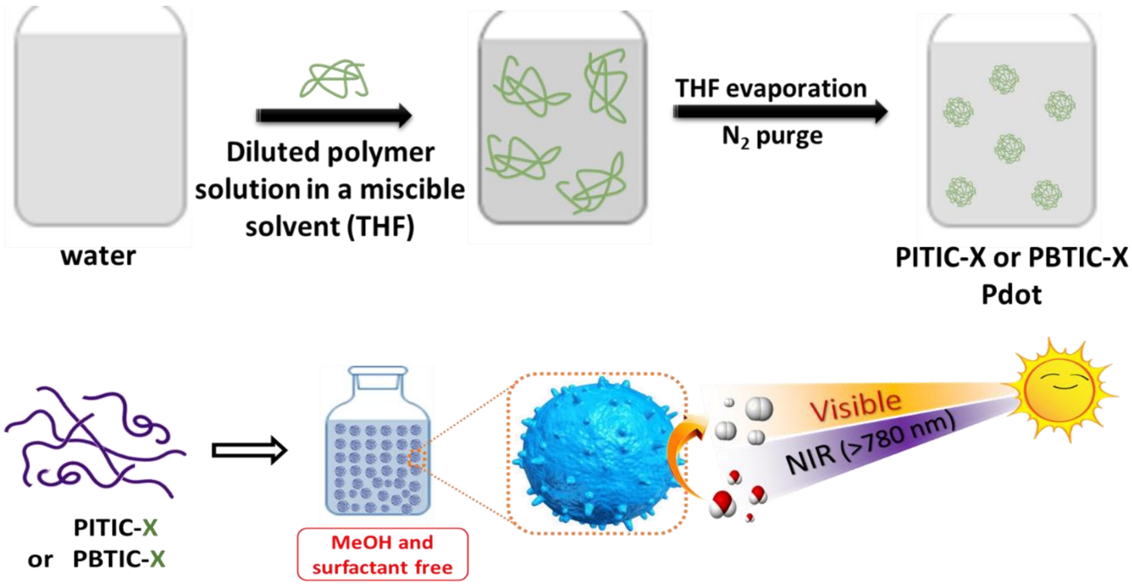

Supplementary Fig. 23. Synthesis of Pdot structure.

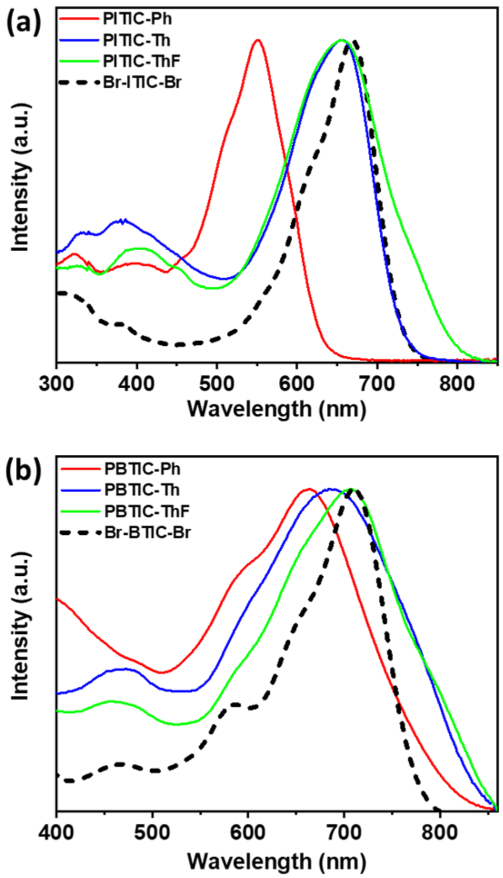

Supplementary Fig. 24. Comparison between the UV-vis absorption of (a) Br-ITIC-Br and (b) Br-BTIC-Br monomers and their polymers with different linkers.

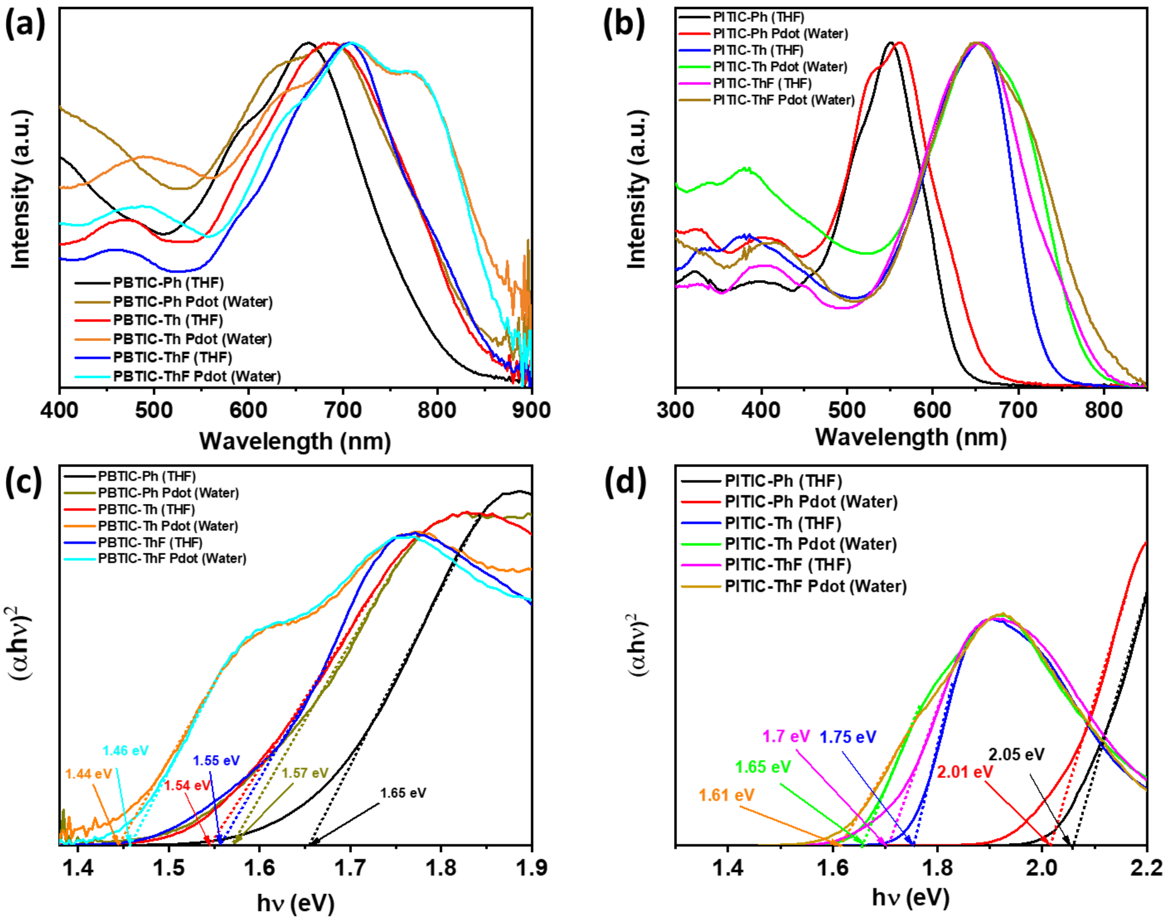

Supplementary Fig. 25. UV-vis absorption spectra of polymers in THF (solid lines) and as Pdot in water solution (dotted lines) for (a) PBTIC-X series and (b) PITIC-X series. The Tauc plot of $(\alpha h\nu)^2$ versus ($h\nu$) from the UV-Vis spectra for calculating the bandgap of (c) PBTIC-X series and (d) PITIC-X series. The optical bandgap (E_g) of the polymers in THF and Pdots in water are extracted from Tauc plots ($(\alpha h\nu)^2$ versus ($h\nu$)) and $\alpha h\nu = A(h\nu - E_g)^{1/2}$ by extrapolation of the linear part of the curve to the energy axis to obtain the optical bandgap.

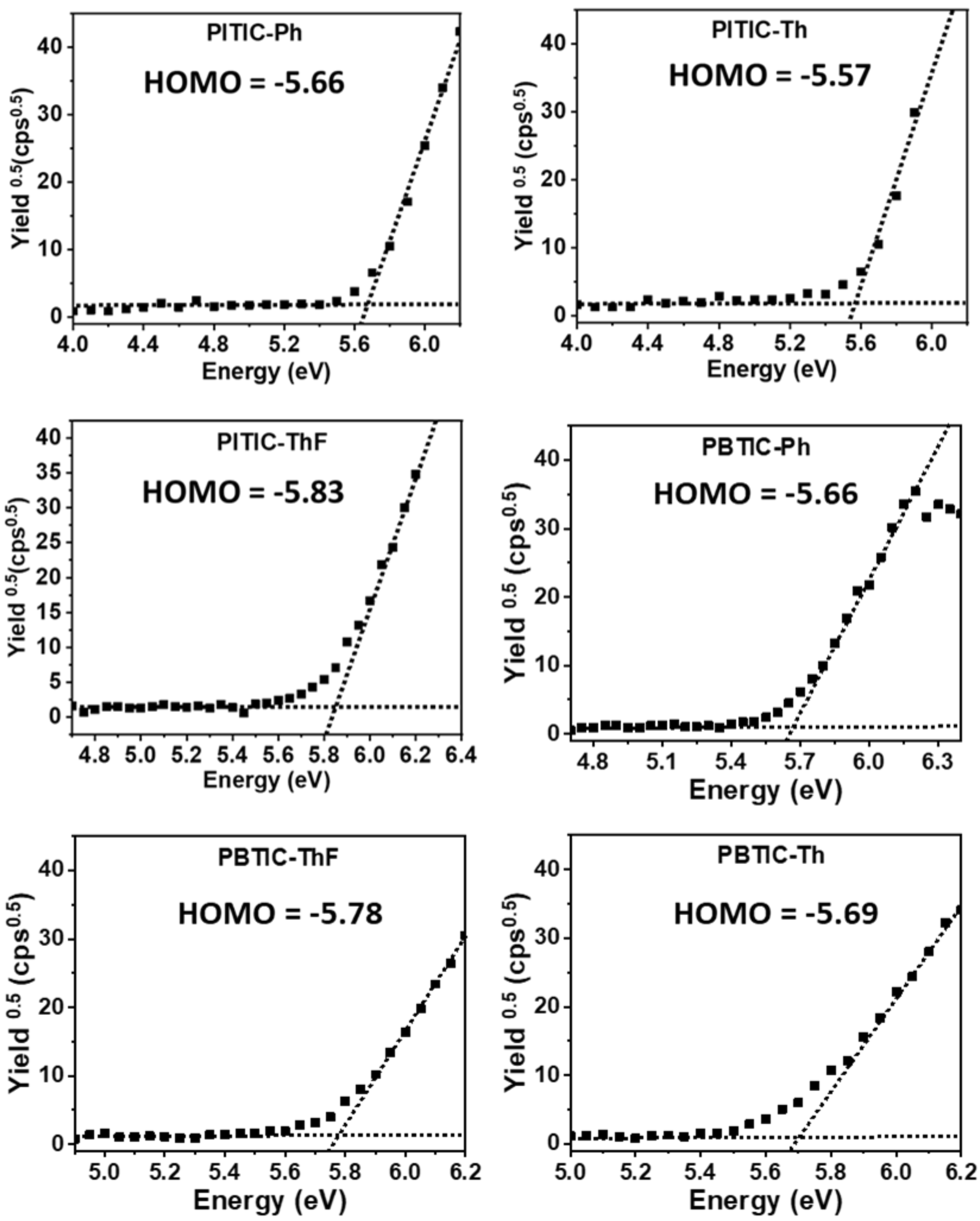

Supplementary Fig. 26. The HOMO levels of six conjugated polymers were determined by a photoelectron spectrometer. The highest occupied molecular orbital (HOMO) energy level of all polymers was determined by photoelectron spectrometry. The energy of the HOMO is subtracted from Eg to obtain the value of the lowest unoccupied molecular orbital (LUMO), and the data are summarized in supplementary **Table 2**.

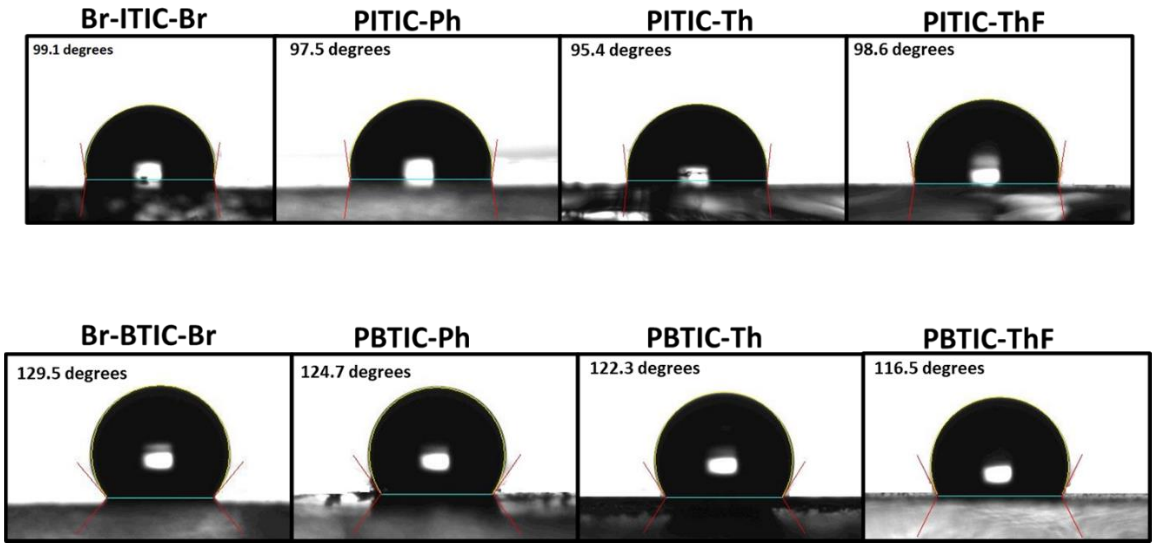

Supplementary Fig. 27. Water contact angles for all polymer materials.

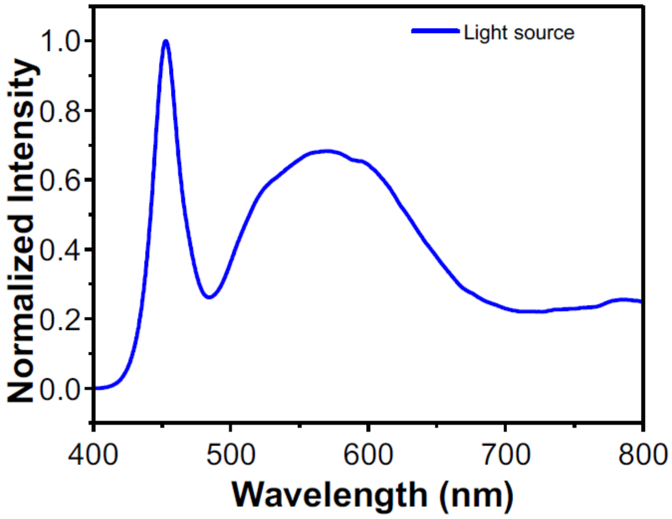

Supplementary Fig. 28. Emission spectra of the light source used in the hydrogen evolution reaction experiment (PAR30 LED lamp, λ > 420 nm, 20 W, 6500 K).

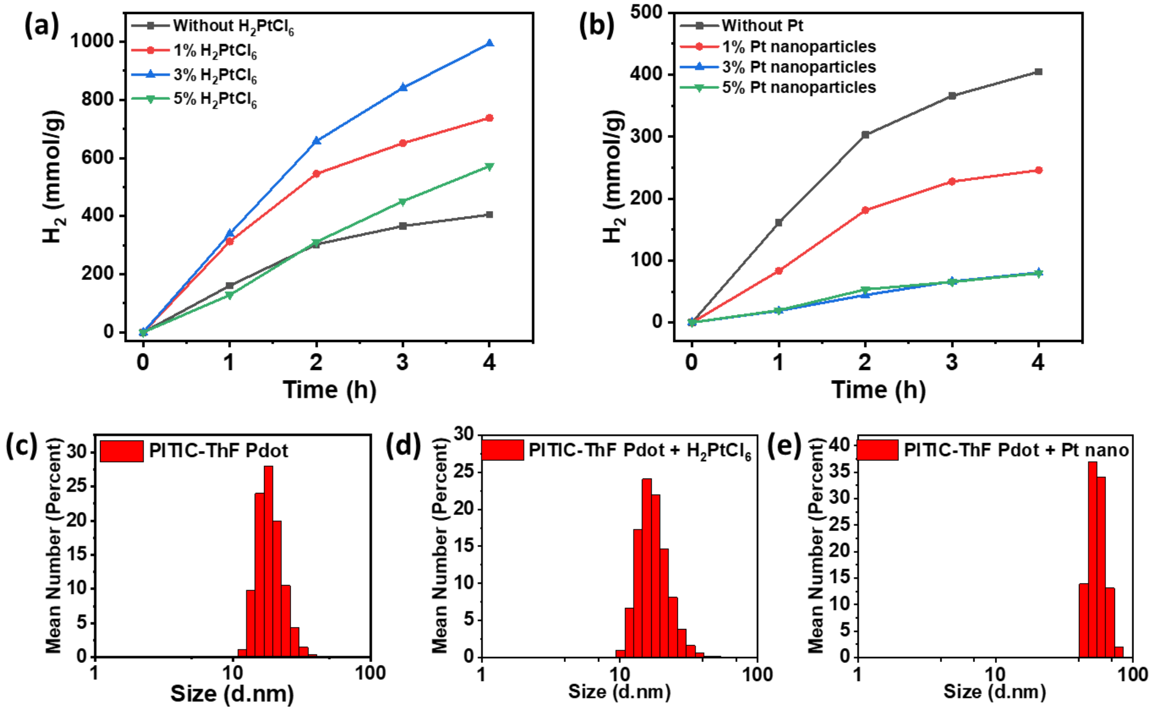

Supplementary Fig. 29. (a) Optimizing the concentration of H_2PtCl_6 during the photocatalytic hydrogen production using PITIC-ThF Pdot. (b) effect of different concentrations of Pt nanoparticles on the HER of PITIC-ThF Pdot. (c), (d), and (e) hydrodynamic diameter spectra of PITIC-ThF Pdot without Pt, in the presence of H_2PtCl_6, and in the presence of Pt nanoparticles, respectively.

Two different types of Pt were tested with the PITIC-ThF Pdots: chloroplatinic acid solution (H_2PtCl_6 in water) and platinum nanoparticles (particle size of 3 nm) dispersed in an aqueous solution. H_2PtCl_6 enhances the HER of the PITIC-ThF Pdots, and the optimized amount of H_2PtCl_6 is 3%, as shown in supplementary Fig. 29a. The Pt nanoparticles decrease the HER of the PITIC-ThF Pdots owing to aggregation (Supplementary Fig. 29b); notably, the particle size of the PITIC-ThF Pdots with the Pt nanoparticles is larger than that with H_2PtCl_6 (Supplementary Figs. 29c, 29d, and 29e).

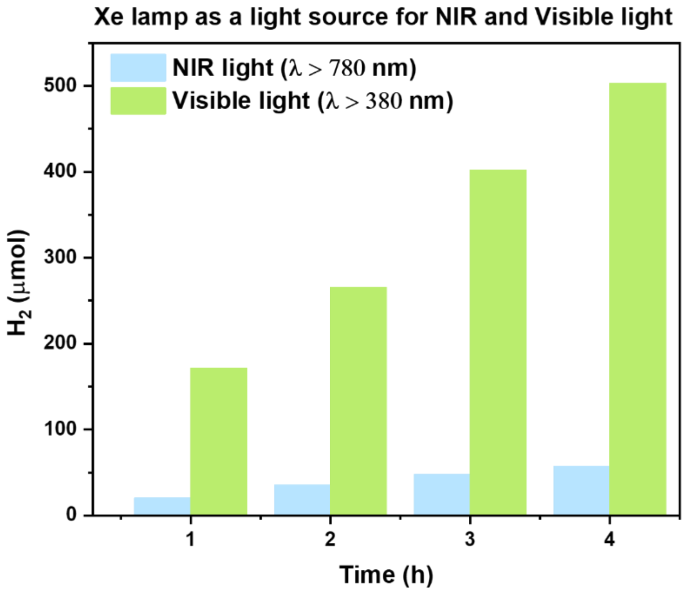

Supplementary Fig. 30. Time course of the produced H_2 for the PITIC-ThF under NIR light and Visible light using the same light source (Xe lamp). We performed two separate tests on the HER of our best material (PITIC-ThF Pdot) using visible and NIR light, respectively, while utilizing the same Xenon lamp as the light source. For both experiments, we dispersed 5 mg of PITIC-ThF Pdot in 10 mL of 0.1M AA solution with 3% Pt as the cocatalyst. A light cut filter at λ>380 nm and a light intensity of 1000W/m^2 (1 sun) were used in the visible light experiment, while in the NIR experiment, a light cut filter at λ>780 nm and a light intensity of 3000W/m^2 were employed. In supplementary Fig. 29, PITIC-ThF Pdot achieved a HER of 171.3 μmol/h under visible light and 20.2 μmol/h under NIR light using the Xenon lamp as the light source.

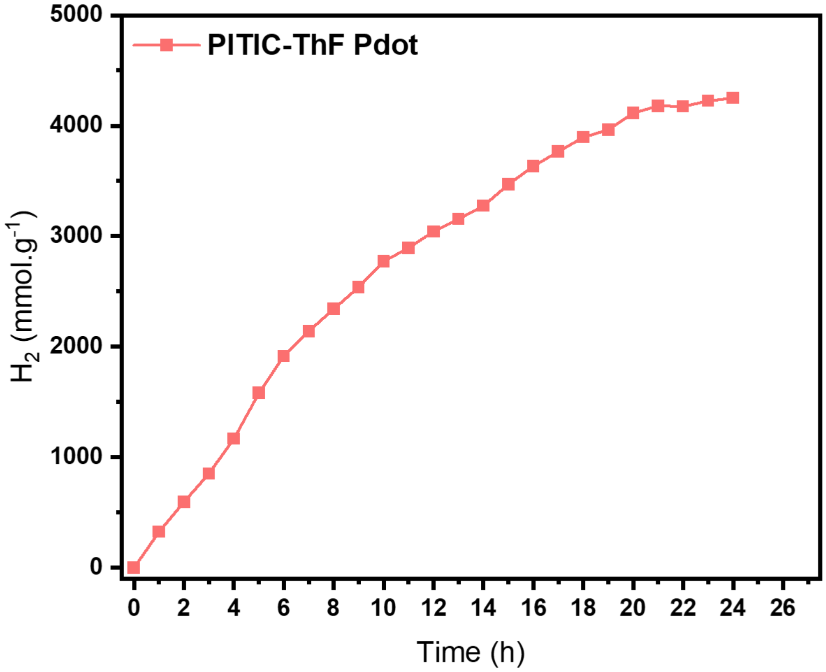

Supplementary Fig. 31. Stability tests. 24 h H_2 evolution stability tests of PITIC-ThF Pdot. Conditions: ascorbic acid (AA, 0.1 M), white LED light (λ > 420 nm, 20 W, and 6500 K), and 3% H_2PtCl_6. The results, as displayed in the figure, revealed a hydrogen production of 4115 mmol. g^{-1} after 20 hours, which then reached a nearly stable state. We posit that the limited time of H_2 production activity (up to 20 hours) can be attributed to the aggregation of polymer nanoparticles during the photocatalytic experiments.

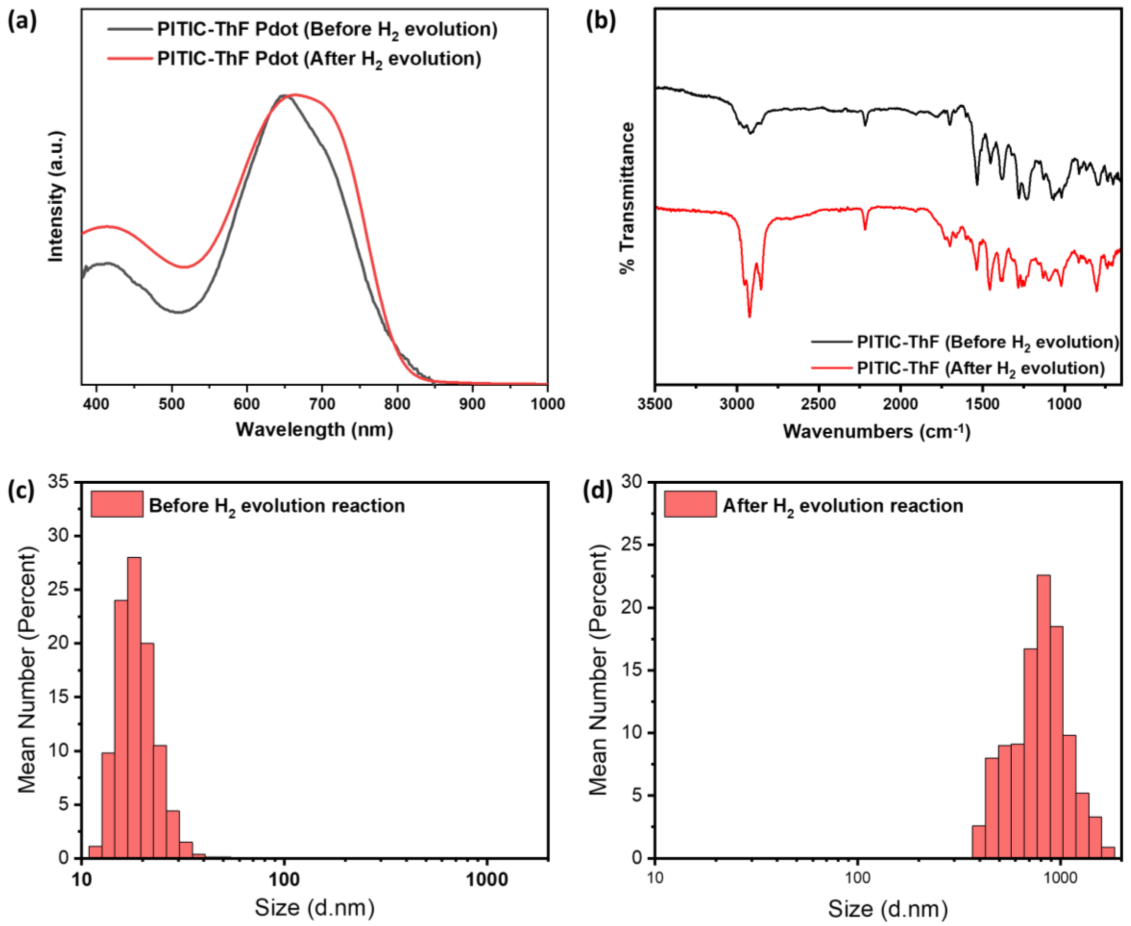

Supplementary Fig. 32. (a) Normalized absorption spectra, and (b) fourier transform infrared (FTIR) before and after 24 h of H_2 evolution of PITIC-ThF Pdot. (c) and (d) the dynamic light scattering (DLS) before and after 24 h of H_2 evolution of PITIC-ThF Pdot, respectively.

To investigate the stability of the best material, PITIC-ThF Pdot, the photocatalysts were subjected to a 24-hour extended stability test (as shown in supplementary Fig. 31). During this test, the photocatalysts were analyzed using various techniques such as Fourier transform infrared spectroscopy (FTIR), dynamic light scattering (DLS), and UV-Vis absorption analysis, both before and after 24 hours of H_2 evolution. The FTIR analysis showed no change before and after the H_2 evolution reaction (as seen in supplementary Fig. 32). However, the UV-Vis absorption analysis showed a slight red shift in the absorption

spectrum of PITIC-ThF Pdot after 24 hours of H_2 evolution (as seen in supplementary Fig. 32a), which suggests that Pdot aggregation may be responsible for the slower reduction in the HER rate. The red shift in the absorption spectrum could be due to the Pdot particles aggregating, as larger particles tend to have a broader absorption spectrum. To confirm this hypothesis, the DLS analysis was conducted. The results showed that the average particle size increased from 30 nm to 1 μm after the H_2 evolution reaction (as seen in supplementary Figs. 32c, and 32d). The increase in particle size indicates that Pdot particles had aggregated, which could be the reason for the slower reduction in the HER rate observed during the stability test.

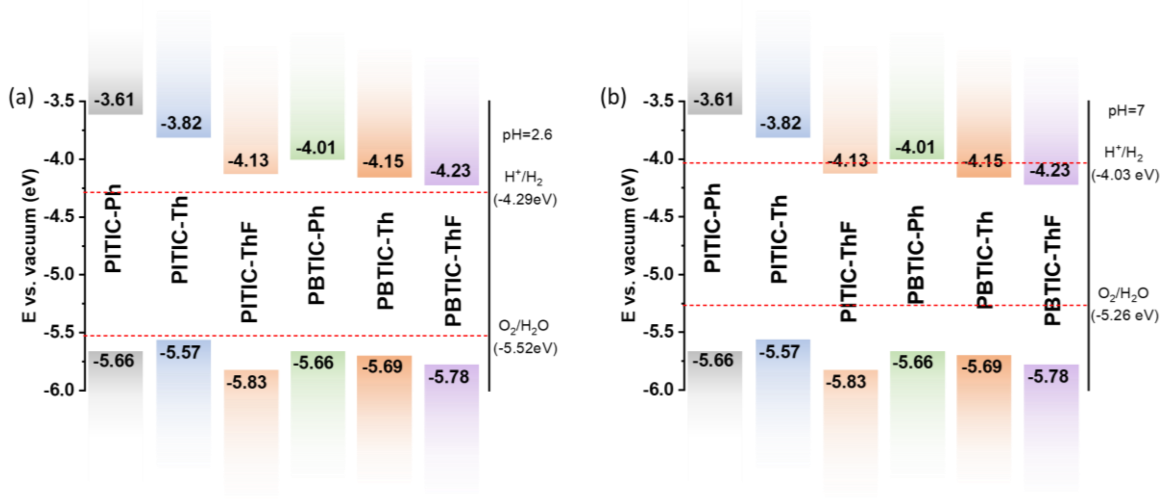

Supplementary Fig. 33. Energy level diagrams of the all-polymers were measured using a photoelectron spectrometer. The dashed lines correspond to the proton reduction potential (H^+/H_2), water oxidation potential (O_2/H_2O) (a) in acidic medium (pH = 2.6), (b) in neutral medium (pH = 7).[1] All energy levels and electrochemical potentials are expressed relative to vacuum (using −4.44 V versus vacuum as equivalent to 0 V versus SHE).[2]

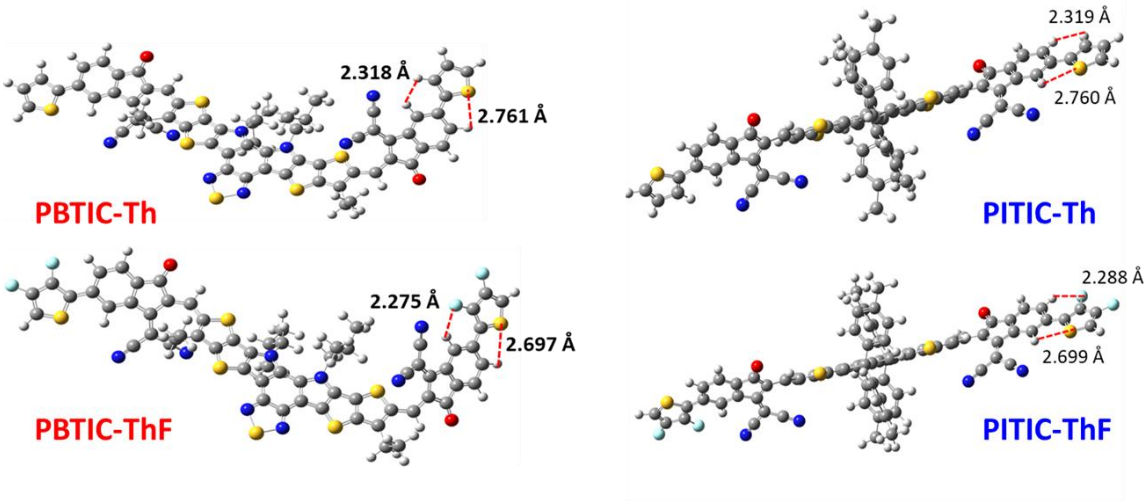

Supplementary Fig. 34. Molecular models of PBTIC-Th, PBTIC-ThF, PITIC-Th, and PITIC-ThF with F−H and S−H bonding distance.

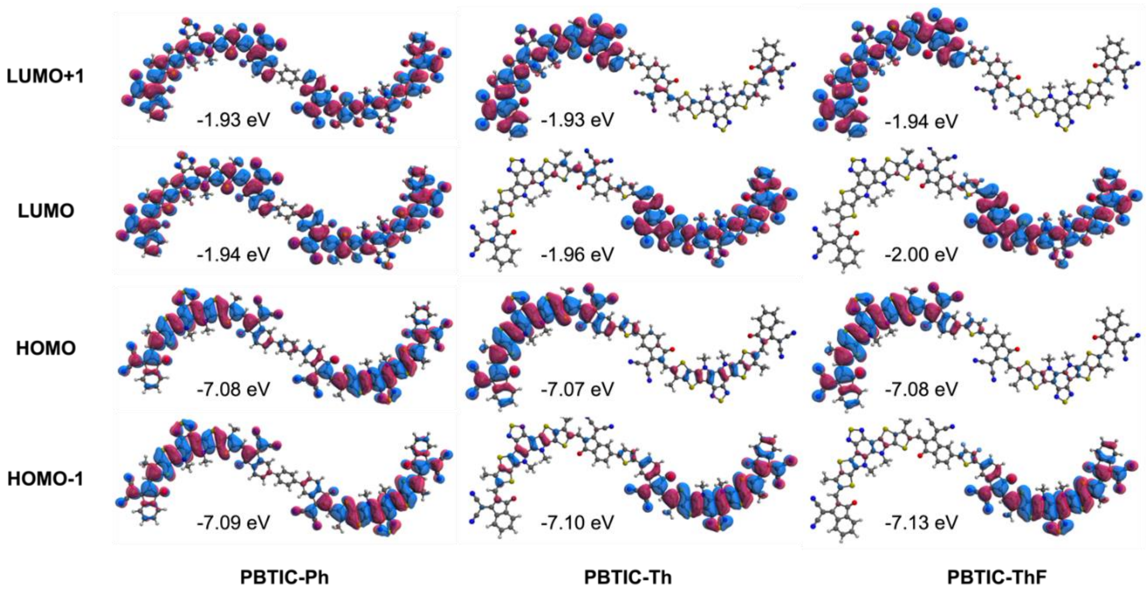

Supplementary Fig. 35. The frontier orbitals (isovalue: 0.015 Å3) of PBTIC series.

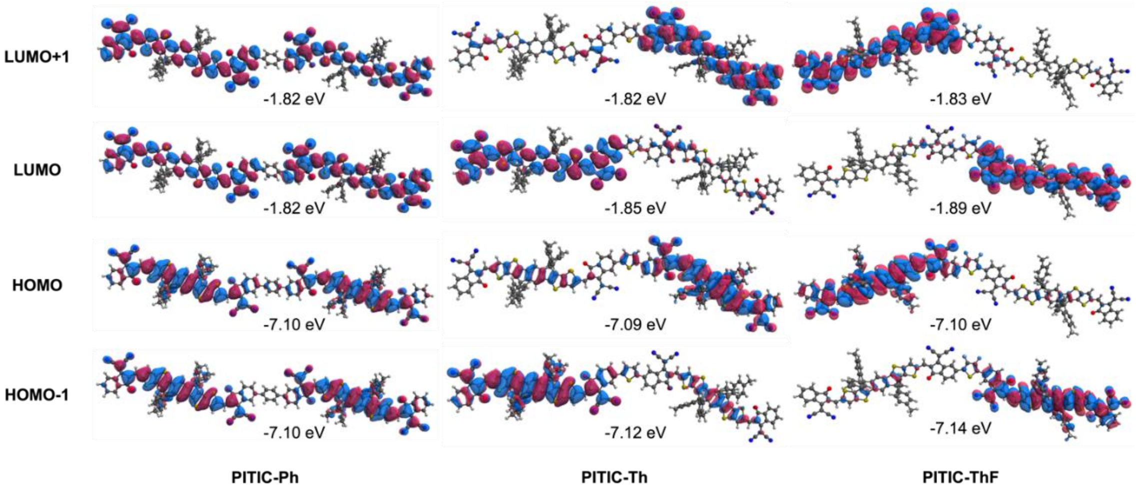

Supplementary Fig. 36. The frontier orbitals (isovalue: 0.015 Å3) of PITIC series.

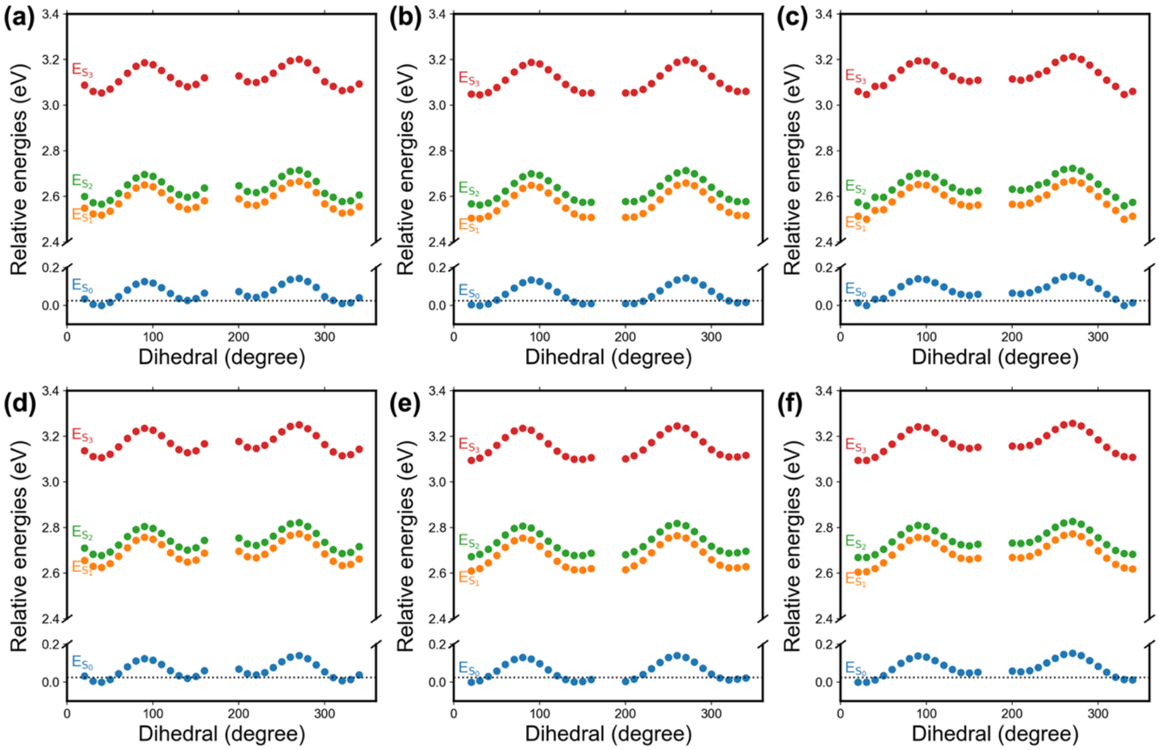

Supplementary Fig. 37. The relative energy levels along the linker-acceptor dihedral angle scanning of ground state (S_0) and excited states (S_1, S_2 and S_3) for (a) PBTIC-Ph, (b) PBTIC-Th, (c) PBTIC-ThF, (d) PITIC-Ph, (e) PITIC-Th, and (f) PITIC-ThF. The dotted line represents the $k_B T$ at the room temperature.

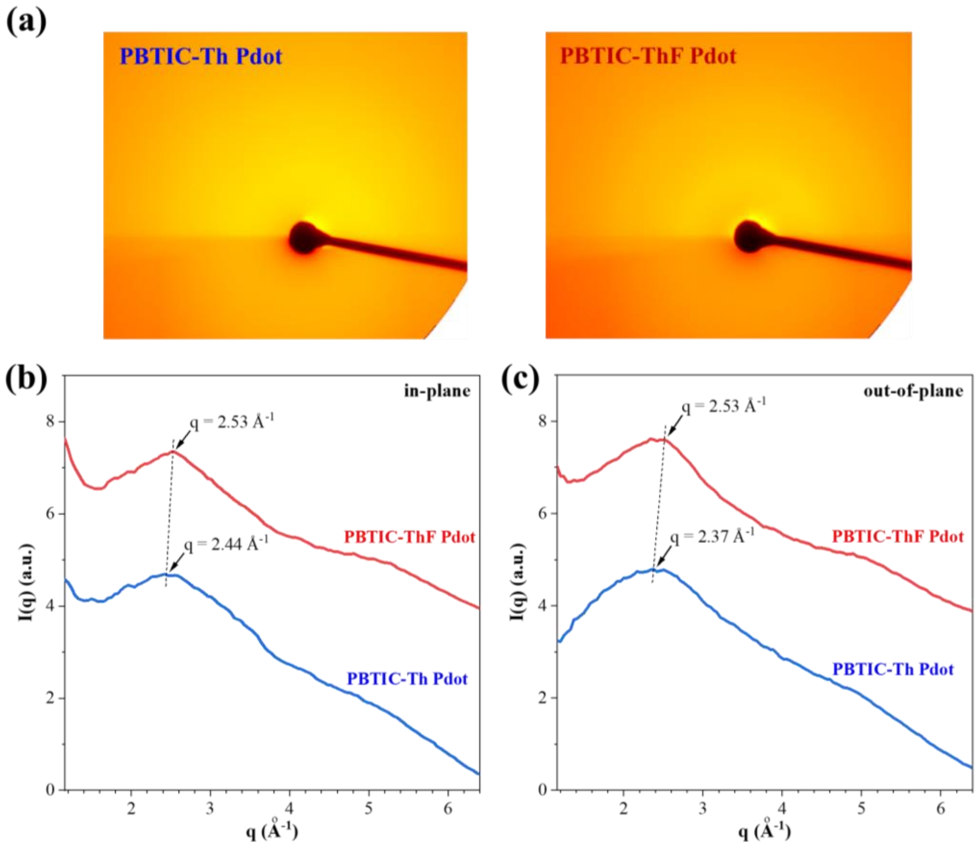

Supplementary Fig. 38. (a) 2D GIWAXS patterns measured for PBTIC-Th Pdot and PBTIC-ThF Pdot thin films spin-coated on Si wafers. The corresponding 1D GIWAXS profiles, including in-plane and out-of-plane scattering profiles ascribed to the scattering with a direction parallel and perpendicular to the thin film plane, respectively, are shown in (b) and (c).

To more understand the role of the dihedral angle in dominating the photophysical property, structural information about its influence on the internal chain segmental packing in PBTIC-Th Pdot and PBTIC-ThF Pdot (taken as model examples) was further examined by the grazing-incidence wide-angle X-ray scattering (GIWAXS), as shown in supplementary Fig. 38. 2D GIWAXS patterns of thin films of the two samples spin-coated on Si wafers displayed isotropic scattering (supplementary Fig. 38a), revealing that the chain segmental packing did not have preferred orientation. As confirmed by the corresponding 1D

GIWAXS data, the in-plane and out-of-plane scattering intensity profiles of each sample exhibited almost the same positions of the diffraction peaks (supplementary Fig. 38b **vs** supplementary Fig. 38c). However, in comparison to the case of PBTIC-Th Pdot, the peak positions observed for PBTIC-ThF Pdot were located at higher q and the diffraction peak at $q = 2.53$ Å^{-1} characteristic of the chain segmental packing became sharper. This observation indicated that the substitution with fluorines to form stronger hydrogen bonds for reducing dihedral angle into the smaller one (i.e., changing from 23.41 to 17.66) effectively improved the ordering along with a reduction in the average spacing of the internal chain segmental packing. That is to say, the improved photophysical properties of PBTIC-ThF Pdot were very likely subjected to the development of a more ordered nanocrystal, mainly endowed with a smaller dihedral angle.

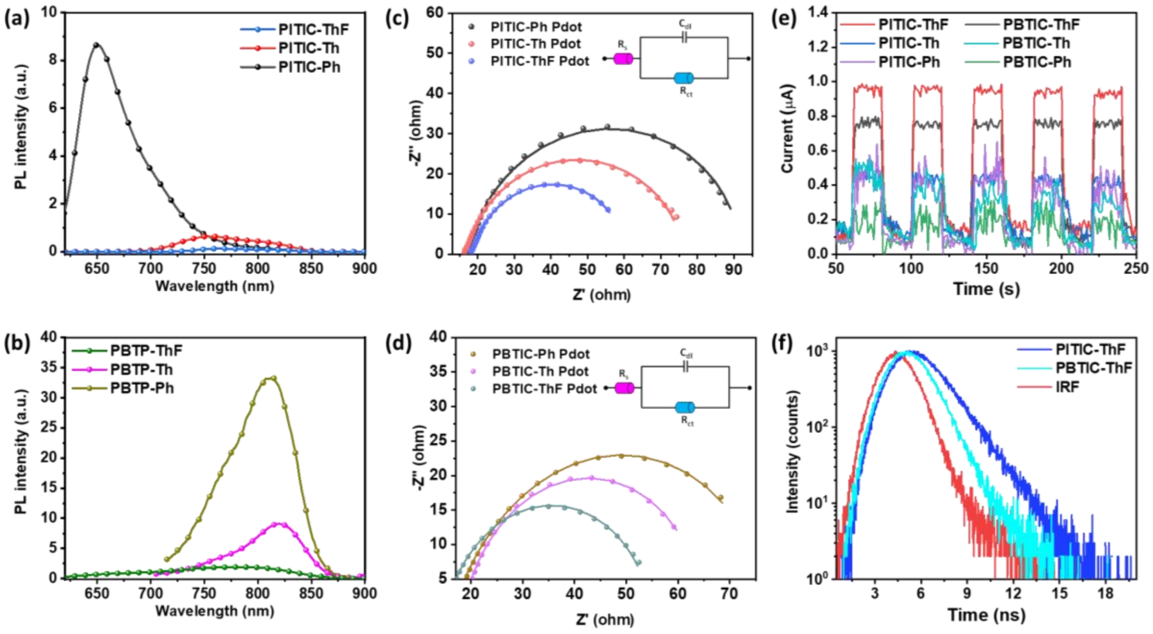

Supplementary Fig. 39. (a) and (b) Photoluminescence emission spectra of PITIC-X and PBTIC-X based Pdots, respectively. (c) and (d) The electrochemical impedance spectroscopy (EIS) of PITIC-X and PBTIC-X based Pdots, respectively. (e) Photocurrent response of presented polymers. (f) Time-resolved decay traces of pristine PITIC-ThF and PBTIC-ThF as solutions in water (excitation wavelength, 600 nm; emission wavelength, 750 and 800 nm, respectively).

The steady-state photoluminescence spectra measurements can explain the charge recombination of the photocatalyst, which strongly affects its photocatalytic activity. Supplementary Figs. 39a and 39b present the photoluminescence (PL) emission of the polymers, and the results exhibit the strongly quenched PL emission of the ThF- and Th-based polymers compared to the Ph-based polymers, revealing lower charge recombination during the photocatalytic reaction. Supplementary Figs. 39c and 39d show the electrochemical impedance spectroscopy (EIS) Nyquist plots of the six polymers dot. The arc radii of the PITIC-ThF and PBTP-ThF Pdots are smaller than those of the

corresponding phenyl- and thiophene-containing comonomers, suggesting a smaller charge transfer resistance in the photocatalytic Pdots (Supplementary Table 9). In addition, supplementary Fig. 39e shows the photoresponse ability of the corresponding samples using the transient photocurrent response, which was collected under visible light irradiation at 1.1 V (vs. Ag/AgCl). The photocurrent-time (I-t) curves for samples were assumed by adopting the switch on-off illumination method. The results show that the PITIC-ThF and PBTIC-ThF Pdots present strong photocurrent responses compared to other Pdots. This is clear evidence that both the PITIC-ThF and PITIC-Th Pdots have the highest photocurrent density, suggesting that they also have the highest charge separation efficiency, which leads to their high photocatalytic performance. Moreover, the time-resolved fluorescence decay spectra of the PITIC-X-based polymers present a longer excited state fluorescent lifetime than that of the PBTIC-X-based polymers (Supplementary Fig. 39f and supplementary Table 2). As a result, the recombination rate of the photogenerated electron-hole pairs with PITIC-X polymers will be slower than that of the PBTIC-X-based polymers, which is favorable for more electron transfer to the cocatalyst for the proceeding reduction reaction.

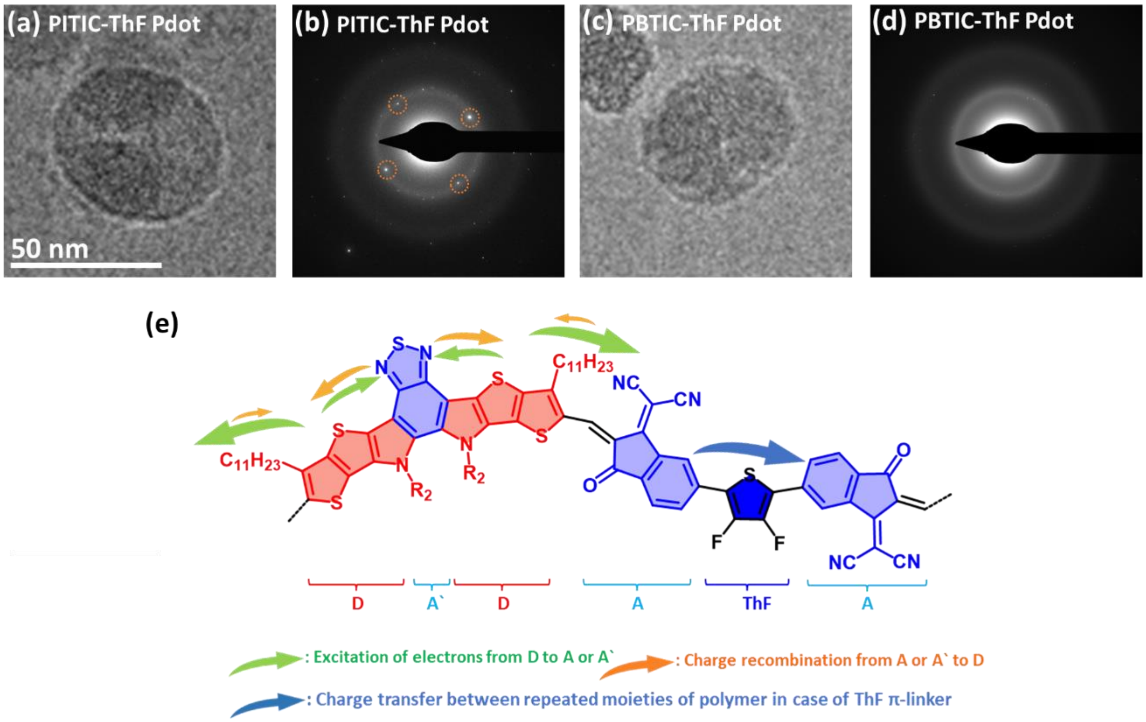

Supplementary Fig. 40. (a) Cryo-TEM micrograph, and (b) cryo-electron diffraction of the PITIC-ThF Pdots. (c) Cryo-TEM micrograph, and (d) cryo-electron diffraction of the PBTIC-ThF Pdots. (e) Schematic diagram showing the charge transfer through the PBTIC-ThF polymer chain during the photocatalytic reaction.

The high-magnification cryo-TEM images clearly show spherical particles with lattice fringes in the PITIC-ThF Pdot image (Supplementary Fig. 40a), which distinguishes the PITIC-ThF Pdots from the PBTIC-ThF Pdots (Supplementary Fig. 40c) in terms of crystallinity. The cryo-electron diffraction (Cryo-ED) of the PITIC-ThF Pdots (Supplementary Fig. 40b) displays clear bright spots compared to the PBTIC-ThF Pdots (Supplementary Fig. 40d), denoting that the PITIC-ThF Pdots have better natural crystallinity than the PBTIC-ThF Pdots, which facilitates charge transfer between the PITIC-ThF Pdots and Pt cocatalyst and increases the final yield of the photocatalytic reaction.[3] Furthermore, the structure design of BTIC moiety (A-DA`D-A) contains A`

between the two D, which enables the photo-excited electrons to distribute between the A and A` then the charge recombination occurs easily from the A` to D (Supplementary Fig. 40e). Consequently, all ITIC-based polymers outperform the BTIC-based polymers in terms of their photocatalytic efficiency for H_2 evolution.

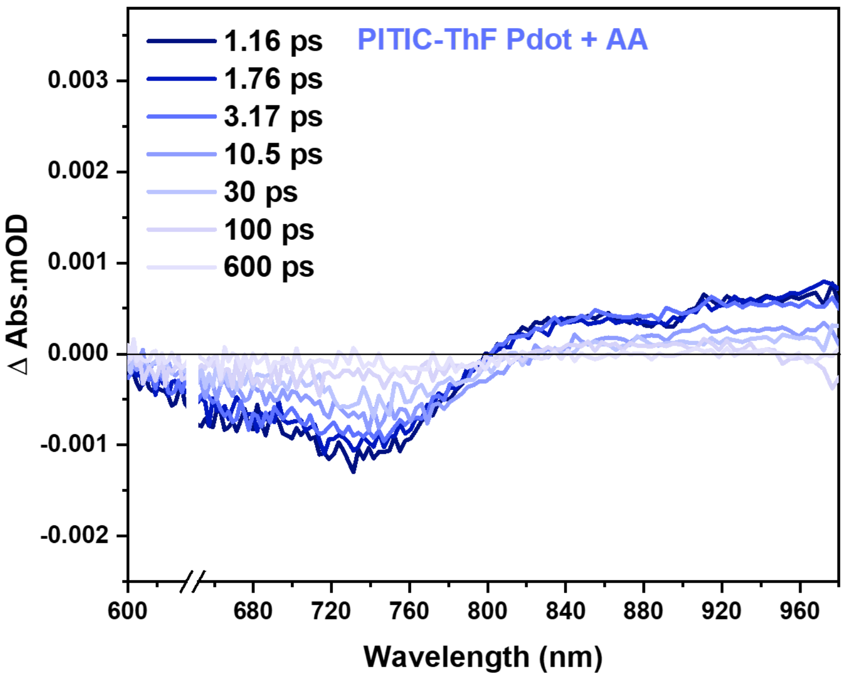

Supplementary Fig. 41. Transient absorption spectra for PITIC-ThF Pdot with 0.1M AA at different time delays after excitation at 650 nm with a power of 0.9 μW.

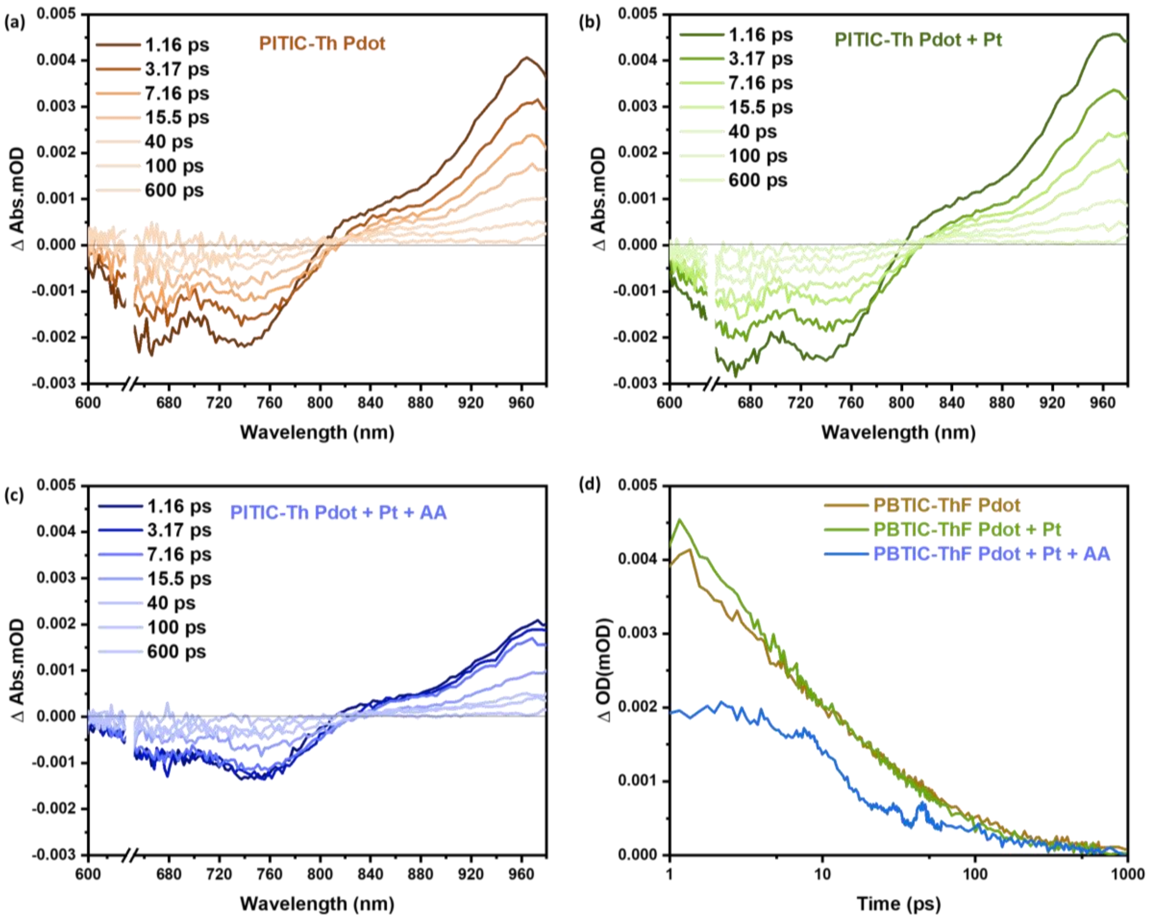

Supplementary Fig. 42. Transient absorption spectra for various samples, including pure PITIC-Th Pdot (a), PITIC-Th Pdot with 3 wt% Pt (b), and PITIC-Th Pdot with 0.1M AA and 3 wt% Pt (c), at different time delays after excitation at 650 nm with a power of 0.9 μW. The decay dynamics of the transient absorption were compared for neat PITIC-Th Pdot, PITIC-Th Pdot + Pt, and PITIC-Th Pdot + AA + Pt, when excited at 650 nm and probed at 964 nm, which were assigned to PITIC-Th Pdot exciton decay (d).

The TAS spectra of PITIC-Th Pdot show a broad ground state bleaching (GSB) between 600 nm and 810 nm, with a maximum peak at 740 nm. Additionally, a photoinduced absorption (PIA) was observed starting from 810 nm and reaching a maximum in the NIR region at 964 nm. This PIA is attributed to the singlet exciton absorption of PITIC-ThF Pdot (Supplementary Fig. 42a). In Supplementary Fig. 42b, the TAS spectra reveals a larger

amplitude with the addition of 3% Pt to the Pdot solution, which is consistent with suppressed bimolecular recombination due to electron transfer to Pt. On the other hand, the addition of AA for Pdot samples with Pt strongly reduced the amplitude in supplementary Fig. 42c, indicating efficient hole extraction in the photocatalytic system. The exciton decay dynamics of neat PITIC-ThF Pdot and the photocatalytic system containing Pt or Pt+AA were compared in supplementary Fig. 42e. It was found that the addition of Pt to PITIC-ThF Pdot resulted in a longer-lived decay transient compared to the neat PITIC-ThF Pdot, which is consistent with slower bimolecular recombination kinetics with Pt. The further addition of AA resulted in an accelerated decay of the PITIC-ThF Pdot absorption, consistent with hole transfer to AA.

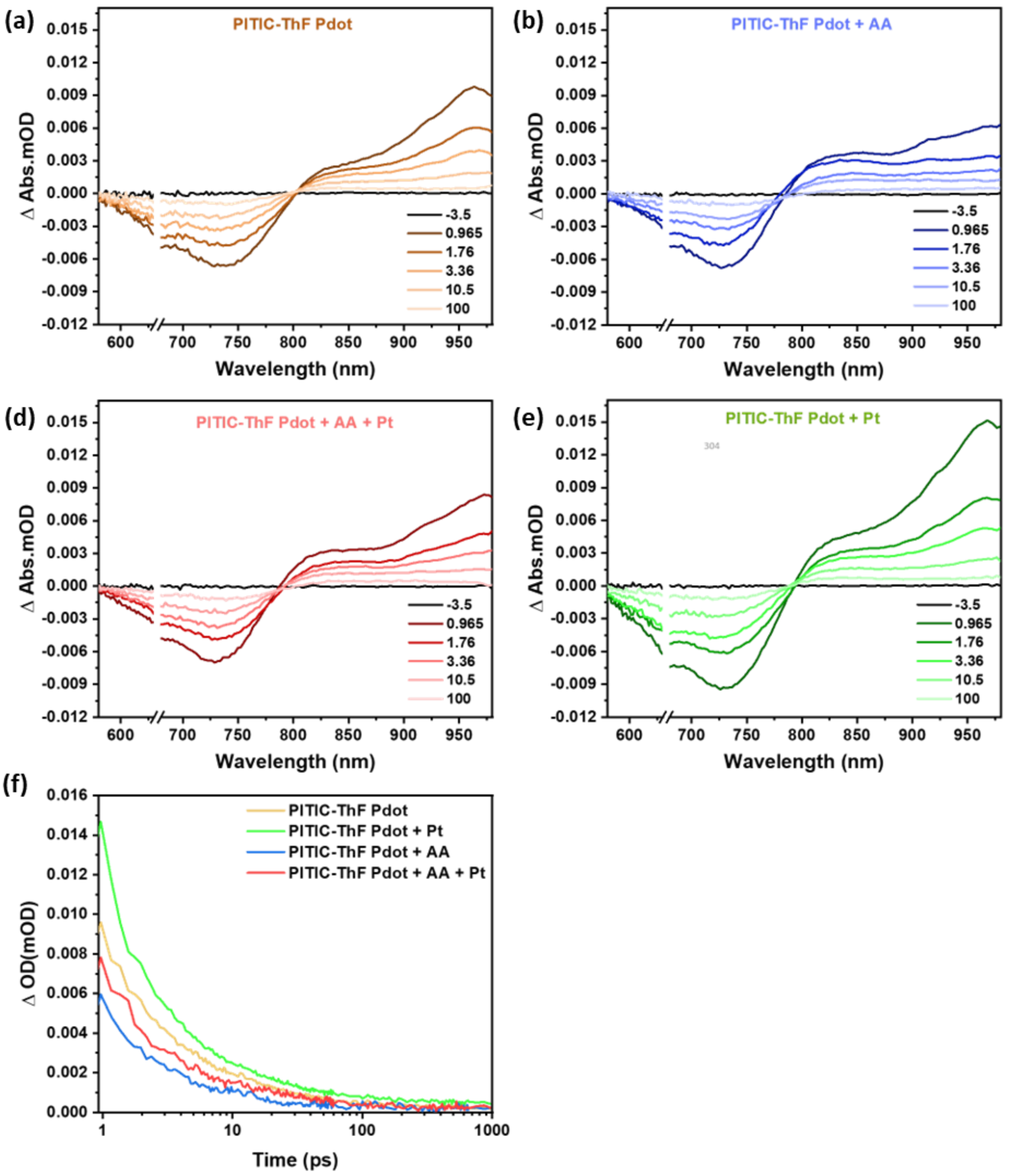

Supplementary Fig. 43. Transient absorption spectra for various samples, including pure PITIC-ThF Pdot (a), PITIC-ThF Pdot with 0.1M AA (b), PITIC-ThF Pdot with 0.1M AA and 3 wt% Pt (c), and PITIC-ThF Pdot with 3 wt% Pt, at different time delays after excitation at 650 nm with a power of 10 μW. The decay dynamics of the transient absorption were compared for neat PITIC-ThF Pdot, PITIC-ThF Pdot + Pt, PITIC-ThF

Pdot + AA, and PITIC-ThF Pdot + AA + Pt, when excited at 650 nm and probed at 959 nm, which were assigned to PITIC-ThF Pdot exciton decay (d).

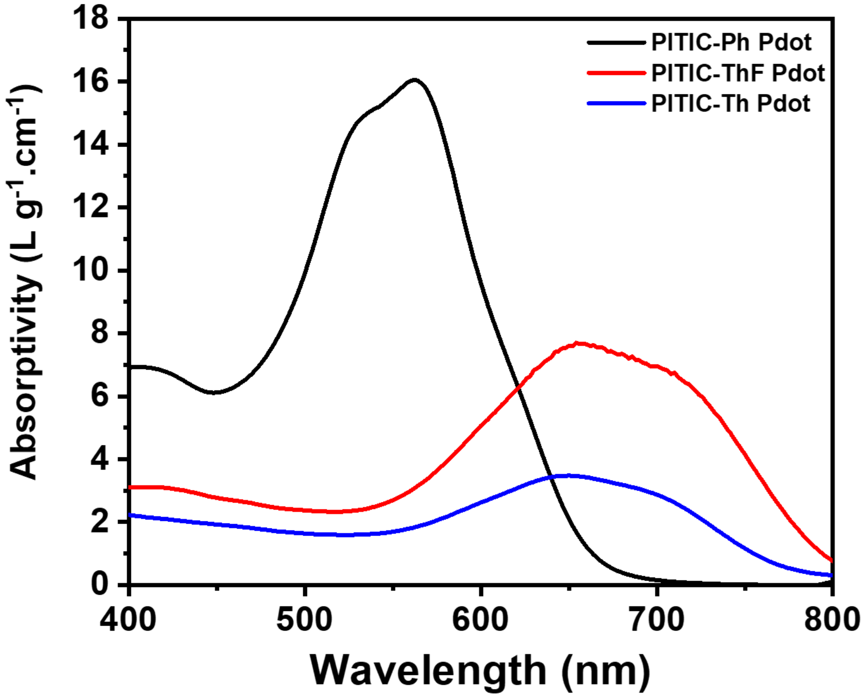

Supplementary Fig. 44. Absorptivity of PITIC-X polymers with different linkers (Ph, Th, and ThF) measured as Pdot in aqueous solution.

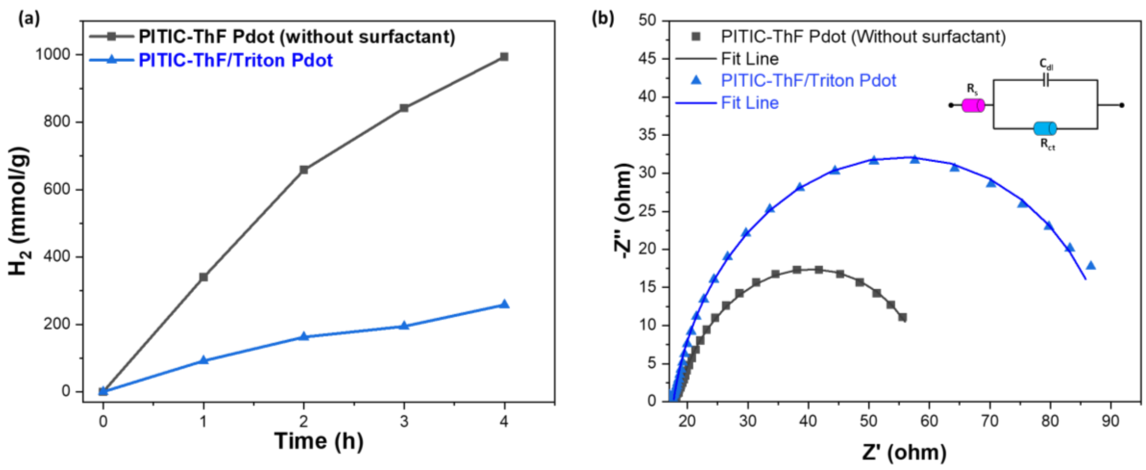

Supplementary Fig. 45. (a) Effect of Triton surfactant on the photocatalytic hydrogen production activity of the PITIC-ThF Pdots. (b) Electrochemical impedance spectroscopy (EIS) of the PITIC-ThF Pdots (with and without Triton surfactant).

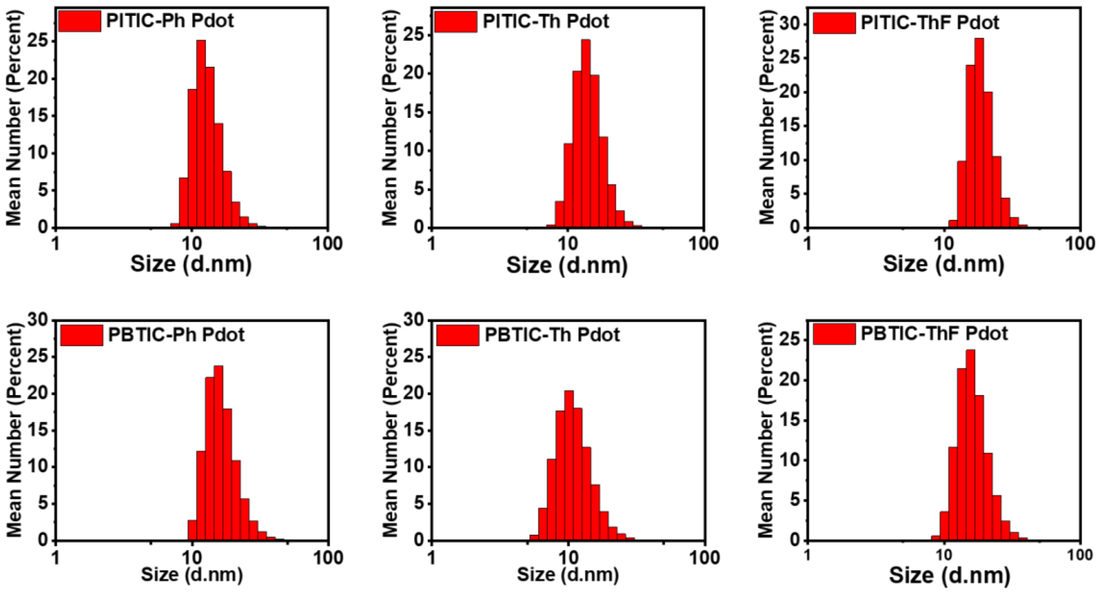

Supplementary Fig. 46. Particle size distributions measured by dynamic light scattering (DLS) of all presented polymers nanoparticles in water.

As shown in supplementary Fig. 46, the particle size distribution of all samples was found to be within a similar range of 5-50 nm. This indicates that the difference in PL intensity observed among different polymer samples (as presented in supplementary Fig. 39) is not likely to be caused by differences in particle size. Instead, it can be attributed to charge separation, which results from the variation in the linker structure within the polymer compositions.

Supplementary Table 1. Physical properties of polymer dots.

Polymer blends	Absorptivity [L.g^{-1}.cm^{-1}]	Average particle size[a] [nm]	T_d (°C)[b]	Contact angle [°][c]
PITIC-Ph	16.1	14.9	293.1	97.5
PITIC-Th	3.8	13.4	297.2	95.4
PITIC-ThF	7.5	15.7	332.1	98.6
PBTIC-Ph	7.5	13.7	413.3	124.7
PBTIC-Th	1.8	11.7	305.8	122.3
PBTIC-ThF	3.1	15.9	274.5	116.5

a) Particle sizes of polymers were determined by DLS methods. b) The decomposition temperature was determined by Thermogravimetric analysis. c) Measured contact angles of polymer films with water at RT.

Supplementary Table 2. Physical and optical properties of the PITIC-X- and PBTIC-X-based polymers

Material	absorption (Water)[nm][a]	HOMO [eV][b]	LUMO [eV][c]	bandgap (water)[eV][d]	bandgap (THF)[eV][d]	Lifetime (ns)[e]	(λ>420nm)HER [mmol h^{-1} g^{-1}][f]	(λ>420nm)HER [μmol h^{-1}][g]	(λ>780nm)HER [μmol h^{-1}g^{-1}][h]	AQY (%)[k] at 420 nm	AQY (%)[l] at 700 nm
PITIC-Ph	530, 561	-5.66	-3.61	2.01	2.05	--	106.2	--	--	--	--
PITIC-Th	647, 690	-5.57	-3.82	1.65	1.75	--	168.7	--	--	--	--
PITIC-ThF	650, 702	-5.83	-4.13	1.61	1.70	1.33	339.7	279	4045	3.9	4.7
PBTIC-Ph	634, 689	-5.66	-4.01	1.57	1.65	--	69.8	--	--	--	--
PBTIC-Th	637, 708, 775	-5.69	-4.15	1.44	1.54	--	121.1	--	--	--	--
PBTIC-ThF	642, 711, 782	-5.78	-4.23	1.45	1.55	0.72	269.4	178	708	2.9	3.1

a) Absorption spectra were measured in water solution. b) Determined by photoelectron spectrometer. c) Derived by extracting the HOMO level from the optical bandgap. d) Derived from the Tauc plots. e) Fluorescence lifetime. f) Conditions: 10 mL of Pdot solution (containing 0.1 mg of the polymer, 0.1 M AA, and 3% H_2PtCl_6), white LED light (λ >420 nm, 20 W, and 6500 K). g) Conditions: 50 mL of Pdot solution (containing 5 mg of the polymer, 0.1 M AA, and 3% H_2PtCl_6), white LED light (λ >420 nm, 20 W, and 6500 K). h) Conditions: 10 mL of Pdot solution (containing 5 mg of the polymer, 0.1 M AA, and 3% H_2PtCl_6), xenon lamp (AM1.5, λ >780 nm, and 3000 W m^{-2}). k) Apparent quantum yield at 420 nm. l) Apparent quantum yield at 700 nm.

Supplementary Table 3. The residual Pd contents were determined by ICP-MS

Polymer	Pd content (wt%)
PITIC-Ph	0.102
PITIC-Th	0.204
PITIC-ThF	0.076
PBTIC-Ph	0.156
PBTIC-Th	0.089
PBTIC-ThF	0.092

Supplementary Table 4. The determined molecular wight of all polymers from GPC data.

Polymer blends	Mw	Mn	PDI (Mw / Mn)
PITIC-Ph	9900	3550	2.79
PITIC-Th	10180	6320	1.61
PITIC-ThF	8950	4320	2.07
PBTIC-Ph	12430	6340	1.96
PBTIC-Th	9270	3610	2.57
PBTIC-ThF	10430	5930	1.75

Supplementary Table 5. Comparative studies of our developed polymer photocatalyst versus other polymer photocatalysts in terms of HER, AQY% with the reaction conditions and light source.

Polymer[a]	Conditions	Light Source	HER (mmol h^{-1} g^{-1})	AQY % at wavelengths (nm)	References
PFBT-Pdots	0.075 mg in 3 mL of 0.2M ascorbic acid.	A LED lamp (λ > 420 nm)	8.3	0.5 at 420 nm	Angew. Chem. Int. Ed. 2016, 55, 12306–12310
PFODTBT Pdot	0.075 mg in 3 mL of 0.2M ascorbic acid.	A LED lamp (λ > 420 nm)	50.0	0.9, 0.3, 0.6, and 0.3 at 420, 500, 550, and 600 nm	Energy Environ. Sci., 2017,10, 1372-1376
PFTFQ-PtPy15	1 mg in 10 mL Water/ 20 vol% TEA	A LED lamp (λ > 420 nm)	12.7	0.4 at 500 nm	ACS Catal. 2018, 8, 7766–7772
F8T2 Pdots/g-C3N4	20 mg in (90 mL Water + 10 mL TEOA)	300 W Xenon lamp (λ > 400 nm)	0.93	5.7, 2.8, and 0.8 at 420, 500, and 550 nm	J. Mater. Chem. A, 2019, 7, 303-311
HE-CP10-Dots	20 mg + 55 mL water + ascorbic acid (1.76 g)	300 W Xenon lamp (λ > 420 nm)	0.84	0.9 at 500 nm	Macromolecules 2019, 52, 11, 4376–4384
F8DTBT Pdots/CNNS	20 mg in (90 mL Water + 10 mL TEOA)	300 W Xenon lamp (λ > 400 nm)	0.181	3.4, 0.4, 0.2, and 0.5 at 420, 500, 550, and 600 nm	Appl. Catal. B: Environ. 2019, 259, 118067
PFN-Br	2.5 mg in (5 mL TEOA + 45 mL water)	300-W Xe lamb (λ > 300 nm)	0.68	0.12, 0.40, 0.44 and 0.19 at 550, 600, 650 and 700 nm	Nano Energy, 2019, 60, 775–783
PBDTBT-7EO (3.0 wt% Pt)	2.5 mg in 50 mL of AA solution (0.2 M)	300-W Xe lamb (λ > 300 nm)	15.9	0.13, 0.14, 0.25, and 0.30 at 420, 500, 550, and 600 nm	iScience, 2019, 13, 33–42.

PFTBTA-PtPy	1 mg in 10 mL Water/ 20 vol% TEA	A LED lamp (λ > 420 nm)	7.34	0.5 at 420 nm	Appl. Catal. B: Environ. 2020, 268, 118436
PFNBTBr Pdots/CNNS	20 mg in (90 mL Water + 10 mL TEOA)	300 W Xe lamp (λ > 400 nm)	1.2	7.71, 2.5, 2.0 at 420, 500, and 550 nm	Appl. Catal. B: Environ. 2020, 270, 118852
PTB7-Th/EH-IDTBR Pdot	2 mg in 20 mL 0.2M AA solution	300-W Xe lamp	28.13	2.0, 2.3, 4.3, 5.6, and 6.2 at 420, 500, 620, 660, and 700 nm	Nat. Mater. 2020, 19, 559–565
PyDTDO-3 (w/o Pt)	1.0 M AA solution / 10 vol% DMF	10 mg in (90 mL of 1M AA + 10 mL DMF)	16.32	3.70, 3.68, 3.93, and 2.30 at 420, 500, 550, and 600 nm	Chem. Sci., 2021,12, 1796-1802.
PS-PEG5-FNP	0.2M AA solution	5 mg in (25 mL ascorbic acid (0.2 M))	37.2	2.5 at 420 nm	Angew. Chem. Int. Ed. 2021, 60, 15590–15597.
PyBS-3	25 mg in (100 mL 0.2M AA solution)	300 W Xenon lamp (λ >300 nm)	100.1	29.3 at 420 nm	Adv. Mater. 2021, 33, 2008498.
ZnCoP-FCP	30 mg in (42.5 mL water + 7.5 mL TEOA)	300 W Xe-lamp with a cutoff filter (λ ≥ 400 nm)	2.76	6.92, 5.19, 5.50, 5.78, 3.17, 1.93 at 400, 450, 500, 550, 700, and 760 nm	Adv. Funct. Mater. 2021, 31, 2009819.
D1/D2/ITIC	0.062 mg in (1.5 mL water + 0.5 mL AA 0.8 M)	LED PAR38 lamp (λ > 420 nm)	60.8	2.2, 4.6, 6.5, 7.1, 6.1, 4.1 at 450, 500, 550, 600, 650, and 700 nm	J. Am. Chem. Soc. 2021, 143, 2875.
gIDTBT:oIDTB R	1 mg in (0.2 M AA (12 mL))	Solar simulator (Asahi Max 303) and an AM1.5g filter	18.5	5.3/1.0/2.9/2.8/0.9% at 400, 440, 620, 660 and 700 nm	Adv. Mater. 2021, 34, 2105007.
PCPDTBSO	2 mg in (1mL NMP + 9 mL ascorbic acid (1M))	300 W Xenon lamp (λ >350 nm)	24.6	0.94, 7.77, 8.72, 4.77, 3.74 at 420, 460, 500, 550, and 600 nm	Appl. Catal. B: Environ. 2021, 298, 120577.

PBDTTS-1SO	6 mg in (3mL NMP + 27 mL ascorbic acid (1M))	300 W Xenon lamp (λ >350 nm)	97.12	13.5, 16.7, 18.5, and 9.8 at 420, 500, 550, and 600 nm	J. Mater. Chem. A, 2022,10, 6641-6648
PM6:PCBM 2:8	1 mg in (0.2 M AA (12 ml))	Solar simulator (Asahi Max 303) and an AM1.5g filter	73.7	8.7, 8.8, 7.7, 6.6, 2.6 at 400, 470, 560, 620 and 700 nm	Nat. Energy. 2022, 7, 340-351
**PBTIC-ThF Pdot**	**0.1 mg in (10 mL 0.1M ascorbic acid)**	**A LED lamp (λ > 420 nm)**	**269.4**	**3.9, 3.2, 3.1, 3.9, and 4.7 at 420, 500, 550, 600 and 700 nm**	**This work**
**PITIC-ThF Pdot**	**0.1 mg in (10 mL 0.1M ascorbic acid)**	**A LED lamp (λ > 420 nm)**	**339.7**	**2.9, 2.7, 2.5, 2.8, and 3.1 at 420, 500, 550, 600 and 700 nm**	**This work**

Supplementary Table 6. Comparative studies of our developed polymer photocatalyst versus other photocatalysts in terms of photocatalytic hydrogen evolution under both visible and NIR light.

Photocatalysts	Conditions	Visible light (>420 nm) (mmol $g^{-1}h^{-1}$)	NIR light (>780 nm) (μmol$g^{-1}h^{-1}$)	References
Au/$La_2Ti_2O_7$	1.5 mg in 5 mL (1:4) methanol-H_2O solution	0.74	300	Angew. Chem. Int. Ed. 2017, 56, 2064–2068
g-C_3N_4-Co-K	50 mg in (15 mL TEOA + 85 mL H_2O).	0.808	470	J. Colloid Interface Sci. 2020, 561, 719–729
WS2@Cu Hybrids	3 mg + 90 mL H_2O + 10 mL Lactic Acid + 1g PEG	64	175 (>750 nm)	Adv. Funct. Mater. 2018, 28, 1804055.
Black Phosphorus / g-C_3N_4	1.5 mg in (1 ml methanol + 4 mL H_2O)	0.427	101	J. Am. Chem. Soc. 2017, 139, 13234−13242
Au / La2Ti2O7	10 mg in (2 mL methanol + 8 mL H_2O)	0.34	180	ACS Catal. 2018, 8, 122−131.
g-C3N4 / Chlorin e6	10 mg in (4 mL TEOA + 16 mL H_2O)	1.275	312.6	Appl. Catal. B: Environ. 2020, 260, 118137.
CuNi / rGO composite	5 mg in (60 mL H_2O + 10 mL lactic acid)	1.787	86	J. Mater. Chem. A, 2017, 5, 22772–22781.
Black Phosphorus / TiO_2	2 mg in (1 mL methanol + 4 mL H_2O)	0.941	200	ACS Catal. 2019, 9, 3618−3626.
$H_{0.53}WO_3$ / CdS – Au	30 mg in (10 mL lactic acid + 90 mL H_2O)	10	158	J. Mater. Chem. A, 2019, 7, 1076–1082.
C/K-doped RPCN	20 mg in (3 mL TEOA + 27 mL)	1.4	140	Adv. Mater. 2021, 33, 2101455.
**PBTIC-ThF Pdot**	**0.1 mg in (10 mL 0.1M ascorbic acid)**	**269.4**	**---**	**This work**
**PBTIC-ThF Pdot**	**5 mg in (10 mL 0.1M ascorbic acid)**	**35.6**	**708**	**This work**
**PITIC-ThF Pdot**	**0.1 mg in (10 mL 0.1M ascorbic acid)**	**339.7**	**---**	**This work**
**PITIC-ThF Pdot**	**5 mg in (10 mL 0.1M ascorbic acid)**	**55.8**	**4045**	**This work**

Supplementary Table 7. The excited-state and charge-transfer properties for each polymer.

	PBTIC-Ph	PBTIC-Th	PBTIC-ThF	PITIC-Ph	PITIC-Th	PITIC-ThF
$\lvert V_{\mathrm{A_1A_2}} \rvert$[a]	2.49	5.37	6.08	2.70	5.44	5.82
E_{S_1}[b]	2.52	2.50	2.50	2.63	2.61	2.61
$\lambda_{S_1 \to S_0}$[c]	131	128	127	158	155	154

[a] The electronic coupling element (in meV) of the electron transfer between the neighboring acceptor sites computed using CDFT.
[b] The S_1 vertical excitation energy (in eV) computed using TD-DFT.
[c] The reorganization energy (in meV) for transition from S_1 to S_0 state computed using DFT and TD-DFT.

Supplementary Table 8. The equivalent circuit fitted results of EIS data in Fig. 7c.

Electrodes	R_s (Ω)	R_{ct} (Ω)	C_{dl} (F)
PITIC-ThF Pdot (without surfactant)	18.18	48.06	9.862×10^{-6}
PITIC-ThF PS-PEGCOOH Pdot	17.2	73.74	9.819×10^{-6}

Supplementary Table 9. The equivalent circuit fitted results of EIS data in supplementary Fig. 45b.

Electrodes	R_s (Ω)	R_{ct} (Ω)	C_{dl} (F)
PITIC-ThF Pdot (without surfactant)	18.18	48.06	9.862×10^{-6}
PITIC-ThF PS-Triton Pdot	17.68	78.28	9.244×10^{-6}

Supplementary Table 10. The equivalent circuit fitted results of EIS data in supplementary Figs. S39c, and S39d.

Electrodes	R_s (Ω)	R_{ct} (Ω)	C_{dl} (F)
PBTIC-Ph Pdot	16.33	71.41	11.56×10^{-6}
PBTIC-Th Pdot	18.27	52.33	9.414×10^{-6}
PBTIC-ThF Pdot	14.91	42.38	9.54×10^{-6}
PITIC-Ph Pdot	17.02	80.99	9.833×10^{-6}
PITIC-Th Pdot	16.4	62.14	7.4×10^{-6}
PITIC-ThF Pdot	18.18	48.06	9.862×10^{-6}

Supplementary References

1. Wang X, *et al.* Sulfone-containing covalent organic frameworks for photocatalytic hydrogen evolution from water. *Nat. Chem.* **10**, 1180-1189 (2018).

2. Kosco J, *et al.* Generation of long-lived charges in organic semiconductor heterojunction nanoparticles for efficient photocatalytic hydrogen evolution. *Nat. Energy* **7**, 340-351 (2022).

3. Liu A, *et al.* Panchromatic Ternary Polymer Dots Involving Sub-Picosecond Energy and Charge Transfer for Efficient and Stable Photocatalytic Hydrogen Evolution. *J. Am. Chem. Soc.* **143**, 2875-2885 (2021).
